# Exploratory Screening and Replication of Urinary Biomarkers of Fruit and Vegetable Intake in Free-living European Children and Adolescents Using Untargeted Metabolomics

**DOI:** 10.1016/j.tjnut.2025.101302

**Published:** 2025-12-29

**Authors:** Li Yuan, Samuel Muli, Jantje Goerdten, Jodi Rattner, Mira Merdas, David Achaintre, Ronja Foraita, Maike Wolters, Stefaan De Henauw, Monica Hunsberger, Inge Huybrechts, Lauren Lissner, Dénes Molnár, Luis A Moreno, Paola Russo, Toomas Veidebaum, Wolfgang Ahrens, Ute Nöthlings, Pekka Keski-Rahkonen, Kolade Oluwagbemigun, Anna Floegel

**Affiliations:** 1 Department Epidemiological Methods and Etiological Research, Leibniz Institute for Prevention Research and Epidemiology - BIPS GmbH, Bremen, Germany; 2 Unit of Nutritional Epidemiology, Department of Nutrition and Food Sciences, University of Bonn, Bonn, Germany; 3 Nutrition and Metabolism Branch, International Agency for Research on Cancer (IARC/WHO), Lyon, France; 4 Department of Public Health and Primary Care, Ghent University, Ghent, Belgium; 5 School of Public Health and Community Medicine, Institute of Medicine, Sahlgrenska Academy, University of Gothenburg, Gothenburg, Sweden; 6 Department of Pediatrics, Medical School, University of Pécs, Pécs, Hungary; 7 GENUD (Growth, Exercise, Nutrition and Development) Research Group, Faculty of Health Sciences, University of Zaragoza, Instituto Agroalimentario de Aragón (IA2) and Instituto de Investigación Sanitaria Aragón (IIS Aragón), Zaragoza, Spain; 8 Consorcio CIBER, M.P. Fisiopatología de la Obesidad y Nutrición (CIBERObn), Instituto de Salud Carlos III (ISCIII), Madrid, Spain; 9 Institute of Food Sciences, National Research Council, Avellino, Italy; 10 National Institute for Health Development, Estonian Centre of Behavioral and Health Sciences, Tallinn, Estonia; 11 Section of Nutrition and Dietetics, Faculty of Agriculture and Food Sciences, Hochschule Neubrandenburg – University of Applied Sciences, Neubrandenburg, Germany

**Keywords:** dietary biomarkers, food intake measurement, IDEFICS/I.Family cohort, observational study, 24h-DR, FFQ

## Abstract

**Background:**

Accurately measuring fruit and vegetable intake is challenging in epidemiological studies, and this difficulty is even greater in children. Biomarkers of fruit and vegetable intake may enhance objective assessment.

**Objectives:**

This exploratory study aimed to screen for potential biomarkers and assess the replicability of previously reported biomarkers associated with fruit and vegetable intake in a free-living population.

**Methods:**

Using an untargeted metabolomics approach, we quantified the molecular features in urine from the Identification and Prevention of Dietary- and Lifestyle-induced Health Effects in Children and Infants (IDEFICS) and I.Family cohort. To explore complementary temporal dimensions of diet-metabolite associations, we examined both short-term and habitual fruit and vegetable intake in parallel. Partial least squares and random forest were applied using the MUVR algorithm (Multivariate Methods with Unbiased Variable Selection in R) to identify molecular features related to fruit and vegetable intake. A linear mixed regression model was then fitted to selected features. In addition, the fruit and vegetable intake-metabolites associations were explored in the Dortmund Nutritional and Anthropometric Longitudinal Designed (DONALD) cohort to replicate analyses from the IDEFICS/I.Family cohort.

**Results:**

Fruit and vegetable intake were significantly related to 59 features. Ten metabolites could be annotated in the IDEFICS/I.Family cohort. We observed positive associations of total vegetable intake with octenoylcarnitine, total fruit intake with 5-hydroxyindoleacetate and D-pantothenic acid, orange intake with ferulic acid 4-O-glucuronide and ferulic acid 4-O-sulfate, potato intake with gentisic acid, and apple and total fruit as well as total vegetable intake with hippuric acid. The positive associations between hippuric acid and apple/total fruit intake were replicated in the DONALD cohort.

**Conclusions:**

This study shows several plausible urinary biomarkers of fruit and vegetable intake in children and adolescents, with the association between hippuric acid and apple and total fruit intake replicated in an independent cohort, and consistent with previous literature.

## Introduction

Dietary assessment in epidemiological studies presents substantial methodological challenges, particularly when studies involve large populations and span multiple centers. Large national and international studies cohorts including the United Kingdom Biobank [1], European Prospective Investigation into Cancer and Nutrition [2], and the United States NHANES [3] implemented food frequency questionnaire (FFQ) and 24-h dietary recalls (24h-DR) as the core dietary assessment tools. Even though they are confronted with potential systematic and random errors at the individual level, they have demonstrated reasonable consistency at the group level.

Food intake biomarkers (FIBs) are proposed to improve and validate self-report measurement due to their inherent properties. So far, FIBs have been widely studied in various foods, for example, whole-grain wheat [4], coffee [5,6], meat [7], fish [8], cocoa [9], wine [10], citrus fruit [11,12], and cereal products [13]. The preferred methods to identify FIB are human intervention study design with small amount of participates [14]. Although, many identified biomarkers are not well validated. It is not clear whether they are sensitive and specific enough to be implemented in a free-living population exposed to complex diets and diverse potential confounders. Therefore, it is important to evaluate the concordance between identified biomarkers from intervention studies and self-reported dietary assessment methods used in large population studies. A good example is proline betaine as a biomarker for citrus fruit intake, which has shown good agreement between observational data and 7-d food records [15]. Notably, most of the existing research has been conducted in adult populations, whereas studies in children and adolescents are still scarce [16,17]. Although, compared with adults, children exhibit distinct gut microbiota composition, with lower microbial diversity and different dominant taxa, which can alter microbial metabolism of polyphenols and other food compounds [18]. In addition, developmental changes in hepatic enzymatic activity and renal function may impact both the biotransformation and excretion rates of diet-derived metabolites [19,20]. These factors suggest that biomarker behavior identified in adults may not directly translate to pediatric populations and support the importance of conducting child-specific studies.

Nevertheless, some large-scale efforts have emerged to explore these younger age groups. The IDEFICS (Identification and Prevention of Dietary- and Lifestyle-induced Health Effects in Children and Infants) and its follow-up I.Family study (Investigating the determinants of food choice, lifestyle and health in European children, adolescents and their parents) is a European prospective cohort study aimed at understanding and preventing diet- and lifestyle-related health issues, particularly obesity, in children and adolescents [21]. Both FFQ and 24h-DR have been validated in previous studies using objective biomarkers in the IDEFICS/I.Family cohort. For example, milk intake assessed via FFQ has been validated against urinary calcium and potassium [22], whereas energy and sugar intakes derived from 24h-DR have been validated using doubly labeled water [23] and urinary sugar excretion [24], respectively.

Given that fruit and vegetable intake is a central component of a healthy and sustainable diet, this study was designed to screen potential biomarkers of fruit and vegetable intake in children and adolescents by linking untargeted metabolomics data with reported intake in the IDEFICS/I.Family cohort. To assess the reproducibility of findings, we further examined these associations in the independent Dortmund Nutritional and Anthropometric Longitudinal Designed (DONALD) cohort.

## Methods

### IDEFICS/I.Family

#### Participants and recruitment

The IDEFICS/I.Family study was the main cohort to identify biomarkers, which have been presented in detail elsewhere [21]. Briefly, a total of 16,230 children aged 2 to 9.9 y from 8 European countries (Italy, Estonia, Belgium, Sweden, Germany, Hungary, and Spain) were enrolled in the baseline survey (W0) conducted between 2007 and 2008. Of these, 11,041 children were reexamined, and an additional 2555 children were newly recruited during the first follow-up wave (W1) between 2009 and 2010. A third examination wave (W2) was carried out within the I.Family project between 2013 and 2014, during which parents and siblings were also invited to participate; this wave included 6055 children from W0 and 1050 newly recruited children from W1.

All children aged 2 to 9.9 y at baseline who had provided urine samples at all 3 time points (W0, W1, and W2), completed at least one 24-h dietary record (24h-DR) at each of these time points, and filled out the Children’s Eating Habits Questionnaire were considered eligible for this study. A random subsample of 599 participants was drawn from the eligible population for further analysis. Sample size calculations were performed a priori based on single time point metabolite data, targeting a statistical power exceeding 80%.

#### Dietary intake assessment

To reflect different temporal dimensions of dietary exposure, we examined both short-term and habitual fruit and vegetable intake. Short-term intake is expected to reflect acute metabolic responses to recent consumption and can therefore be more sensitive to detect transient exposure biomarkers. Habitual intake, in contrast, provides information on longer-term dietary behavior and is the measure of choice in most observational studies. Including both short-term and habitual intake in parallel allowed us to explore both transient and sustained associations between diet and urinary metabolite profiles. Short-term intake was defined as 24h-DR collected 1 or 2 d before the urine collection. Thus, only 444 study samples were eligible for the short-term intake in total from all 3 time points (W0: 116; W1: 105; W2: 223). We selected the apple, orange, and potato as short-term intake groups. A full description of the 24-h DR and its validation can be found elsewhere [23,24,25,26]. Habitual intake of total fruit and total vegetables were estimated according to the National Cancer Institute method [27]. Fruit and vegetables groups defined from the 24h-DR and FFQ were grouped and analyzed according to Kipnis et al. [28]. Samples with total estimated energy intake <500 kcal were excluded from the analysis in 24h-DR. The computation of the habitual dietary intake was stratified by sex, whereas adjusted for age and BMI *z*-score. The final habitual intake study sample was 1788 in total from all 3 time points (W0: 597; W1: 596; W2: 595). The study design and validation of FFQ were previously publishes [22,[29], [30], [31]].

#### Urine sample collection

In the IDEFICS/I.Family cohort, urine samples were collected following a standardized protocol across all study centers [32]. Morning urine was collected at home by primary caregivers immediately after the child woke up, using a kit and instructions provided in advance. If transportation to the study center exceeded 2 h, samples were kept refrigerated. Upon arrival, samples were stored at −20°C and then regularly shipped on dry ice to the central biorepository for long-term storage at −20°C [33].

### Dortmund nutritional and anthropometric longitudinal designed

#### Participants and recruitment

For the replication purpose, dietary data and urine samples from the DONALD cohort study were used. Details on recruitment, study design, and methods have been published previously [34]. The DONALD study is an ongoing open cohort that has enrolled 2375 participants between 1985 and 2022. Participants have been followed up from infancy (starting at 3 mo of age) into adulthood, with repeated assessments of dietary intake, health status, and developmental parameters. For the present study, participants were selected based solely on data availability. A total of 297 children aged between 3 and 10.5 y were selected from the DONALD cohort. Of these, 270 had repeated measurements of both dietary intake and urine samples, whereas 27 had data available from a single time point.

#### Dietary intake assessment

Dietary intake was assessed using 3-day weighed dietary records (3d-WDR) at regular intervals. Caregivers (or participants and depending on age) were instructed to weigh all foods and beverages consumed and record them in structured diaries [34]. Dietitians reviewed the records for completeness and plausibility.

#### Urine sample collection

From age 3 y onward, 24-h urine samples were collected during each examination interval. Urine was collected at home by caregivers on the third day of the 3d-WDR, and all biospecimens were stored at the DONALD study center under standardized conditions.

### Ethics statement

The conduct of the IDEFICS/I.Family and DONALD cohorts adhered to the standards of the Declaration of Helsinki. IDEFICS/I.Family can be found at the International Standard Randomised Controlled Trial Number (ISRCTN) Registry under the ID ISRCTN62310987. The protocol was approved by the appropriate ethics committees from each of the 8 study centers. Before enrolling into the study, both the children and their parents gave written informed consent, except for younger children who gave oral consent in addition to their parents’ written consent. DONALD was approved by the Ethics Committee of the University of Bonn (ethics numbers: 098/06 and 185/20). All examinations were conducted with written informed consent from study participants themselves (≥16 y) or their parents.

### Urine sample preparation and metabolomic profiling

Morning urine samples of 599 eligible children and adolescents collected from each of the 3 time points (*N* = 1788; 9 metabolomics data were unavailable due to technical reasons) in IDEFICS/I.Family cohort and 24-h urine of 297 participants from baseline and follow-up (*n* = 567, of whom 270 had repeated urine measurement) in DONALD cohort were used for metabolomic profiling by a system consisted of a Dionex UltiMate 3000 Binary LC system, and a Q-Exactive mass spectrometer with heated electrospray ionization (Thermo Scientific). This untargeted metabolomics procedure was performed at IARC, Lyon, France. Urine samples were first normalized to the lowest specific gravity (1.008). Then, urine samples were mixed with cold acetonitrile, and the supernatant was extracted for analysis. Quality control (QC) samples were a mixing small aliquot of all urine samples. Blank samples were also prepared along with the urine samples in an identical manner, only leaving out urine in the process. Each well plate included 4 individually prepared QCs and 2 blanks; 2 μL samples were injected into UPLC equipped with an ACQUITY UHPLC HSS T3 column (2.1 × 100 mm, 1.8 μm; Waters) held at 45°C. The mobile phase consisted of ultrapure water and liquid chromatography–mass spectrometry (MS) grade methanol, both with 0.05% (vol/vol) of formic acid. The gradient profile was as follows—0 to 6 min: 5% to 100% methanol; 6 to 10.5 min: 100% methanol; and 10.5 to 13 min: 5% methanol. The flow rate was 0.4 mL/min. Mass detection was carried out in a positive/negative switching polarity using the following condition: spray voltage, 4.0 kV; sheath gas, flow rate 50 [arbitrary unit (AU)]; auxiliary gas flow rate, 13 AU; sweep gas flow rate, 3 AU; auxiliary gas heater temperature, 425°C; capillary temperature, 260°C; and a S-Lens RF level, 60%. A full MS scan mode over a mass range of 66.7 to 1000 Da, at a resolution 35,000 with an associated scan rate at 2.1 Hz. AGC target 1e6 and a maximum injection time of 50 ms was applied. Preprocessing of raw data from UHPLC-QE-MS was performed using Compound Discoverer 3.3 software (Thermo Fisher Scientific) to obtain a list of peak intensity, retention times (RTs), and *m*/*z* in negative and positive ionization modes. This procedure generated 2 data sets for the untargeted metabolomics containing 16,559 and 9050 features in positive ionization mode and 11,397 and 5777 features in negative ionization mode in IDEFICS/I.Family and DONALD cohorts, respectively.

### Feature identification process

Molecular features associated with fruit and vegetable intake were selected by multiple statistical approaches first and then sent to IARC for identification. Selected features were identified based on the IARC in-house database of analytical standards with 10 ppm molecular weight and 0.25 min RT tolerances and search of the *m*/*z* values against the Human Metabolome Database [35] with a 10-ppm mass tolerance, considering [M + H]^+^, [M + Na]^+^, and [M − H_2_O + H]^+^ adducts in the positive mode and [M − H]^−^, [M + FA − H]^−^, and [M − H_2_O − H]^−^ in the negative mode. When standards were not available, MS/MS spectra were compared against online spectral databases mzCloud (www.mzcloud.org) or METLIN (www.metlin.scripps.edu) [36] for level 2. The level of identification confidence was determined as proposed by Sumner et al. [37]. Detailed information for analytical conditions, parameters applied for data preprocessing, and QC results are presented in Supplementary Material.

### Statistical analyses

All statistical analyses were performed using R software (version 4.3.2; R Core Team). Subject characteristics were stratified by composite and individual fruit and vegetable intake groups. Continuous values are presented in median ± IQR and categorical data as counts (percentages).

MS features with missing values were excluded using a 2-step procedure. First, features with missing values of >95% were removed from all subjects. Then, features that were unavailable to >30% of consumers were removed. For each batch, missing metabolite intensities were imputed as half of the lowest observed feature intensity, assuming missingness due to detection limits. Feature intensities were log-transformed and *z*-standardized before further analysis.

To identify a minimal subset of molecular features associated with fruit and vegetable intake, the minimally unbiased variable selection algorithm (MUVR; MUVR Package in R) [38] was applied to the complete untargeted metabolomics dataset. In brief, MUVR is a statistical validation framework that integrates recursive variable selection within a repeated double cross-validation scheme. The main steps are as follows: *1*) randomly divided the entire data set into several outer segments, with 1 segment held out as the test set and the remaining data forming the inner set; *2*) further subdividing the inner data into inner segments, with 1 used as a validation set and the rest as a training set, on which partial least squares (PLS) or random forest (RF) models are fitted and tuned using the validation set; *3*) ranking variable importance across the inner segments and recursively eliminating a proportion of the lowest-ranking variables; *4*) the optimum model, trained on the full inner data, is used to predict the held-out outer test set; and *5*) repeating the entire procedure (outer segmentation, inner training/validation, recursive selection, and testing) multiple times, with results averaged to provide robust estimates of model performance and stable sets of selected variables.

In our study, fruit and vegetable intake was treated as response variable and MS features as predictor. Short-term intake was dichotomized (0 = nonconsumers, 1 = consumers), given the large proportion of nonconsumers, and modeled using RF and PLS classification analysis. In contrast, habitual intake was applied as continuous variable in RF and PLS regression analyses. In accordance to [38], we set the modeling parameters as follows: nOuter = 8 (number of outer cross-validation segment); nRep = 100 (number of model repetitions); and varRatio = 0.85 (proportion of variables maintained in the data per model iteration during variable elimination). Model performance was evaluated using percentage of consumer misclassification for dichotomous outcomes and the coefficient of determination (*R*^2^) for continuous outcomes.

Permutation testing was also performed to further evaluate modeling performance. The modeling parameters were the same as those used for the MUVR models outlined earlier (nOuter = 8; varRatio = 0.85), the number of repetitions was 100. Student *t* test was performed using the MUVR function pPerm to establish the statistical significance of differences in model performance between the actual and permuted models [39]. Owing to the computational intensity of the procedure, 1 permutation run was performed per food group.

**FIGURE 1:**
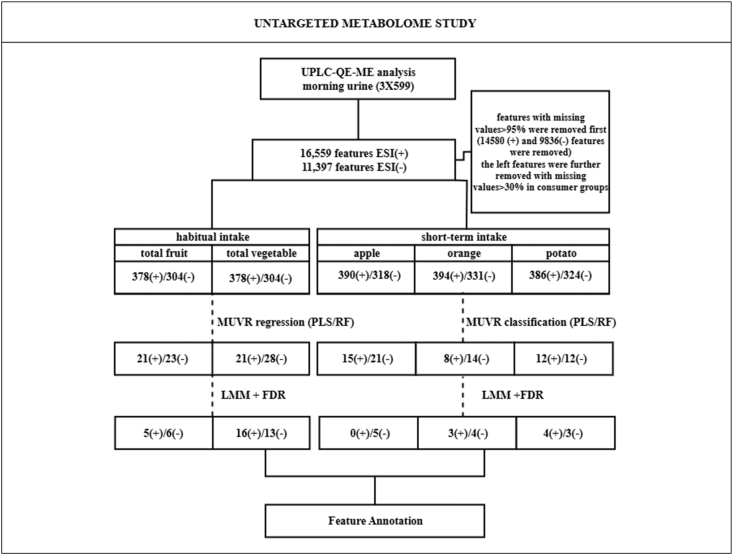
Analysis workflow for discovery of biomarkers of fruit and vegetable intake in IDEFICS/I.Family. ESI (+), positive ionization mode; ESI (−), negative ionization mode; ESI, electrospray ionization; FDR, false discovery rate; IDEFICS, Identification and Prevention of Dietary- and Lifestyle-induced Health Effects in Children and Infants; PLS, partial least squares; RF, random forest.

The features selected by both PLS and RF were analyzed using linear mixed-effects regression models (lme4 package in R) to examine their association with fruit and vegetable intake. Each feature (dependent variable) was regressed on the fruit and vegetable intake variable (independent variable) Separate models were fitted for each metabolite. All models were adjusted for age, sex, country, age-specific and sex-specific BMI *z*-scores [40], energy intake, and analytical batch by including them as fixed effects. Participant ID was included as a random effect to account for within-subject repeated measures. To correct for multiple testing, false discovery rate (FDR) adjustment was performed using the Benjamini-Hochberg procedure on all linear mixed models tested for each intake type. An FDR-adjusted *P* value of <0.05 was considered statistically significant. The analysis workflow of IDEFICS/I.Family is presented in Figure 1.

In a sensitivity analysis, the examination wave (W0, W1, W2) was additionally included as a fixed effect to account for developmental changes over time. The replication study uses dietary intake and urine samples of 2 time points that were selected from the DONALD cohort. Although the IDEFICS/I.Family cohort included 3 time points, the same analysis workflow was applied in DONALD with only minor adaptations to account for differences in data structure. This allowed for a meaningful comparison and facilitated the replication of findings across both cohorts (presented in Supplementary Material).

To evaluate the discriminatory ability of potential biomarkers for distinguishing higher compared with lower intake, an receiver operating characteristic (ROC) analysis was performed. In this approach, the feature intensity was used directly as the predictor, and fruit and vegetable intake was dichotomized into high and low consumers based on the median intake distribution.

### Validation of potential biomarkers for fruit and vegetable intake

A set of validation criteria proposed by Dragsted et al. [41] was applied to evaluate the level of validation of potential biomarkers. The validation scheme was based on 8 questions that included biological and analytical aspects (plausibility, time-response, dose-response, robustness, reliability, stability, analytical performance, and reproducibility).

## Results

### Basic characteristics of participants

In total, 444 and 1788 samples were eligible for short-term and habitual fruit and vegetable intake in the IDEFICS/I.Family cohort, respectively. Among short-term intake samples 147, 71, and 129 consumers were found for apple, orange, and potato intake in the available 24h-DR, respectively. The basic characteristics of the children and adolescents, grouped by fruit and vegetable intake groups are presented in Table 1 [42]. Age, sex, BMI *z*-score, and energy intake distribution did not differ significantly between the groups. Habitual fruit intake was higher than vegetable intake. Available samples for short-term intake were mainly from Sweden and Germany, whereas habitual intake data derived mainly from Italy and Estonia. Within the subsets from IDEFICS/I.Family and DONALD cohort, age, BMI *z*-score, and sex distribution were broadly comparable. Children in the DONALD subset reported higher fruit intake (279 g/d) than those in the IDEFICS/I.Family subset (165 g/d), whereas vegetable intake was higher in IDEFICS/I.Family (110 compared with 70 g/d in DONALD). Descriptive characteristics of the DONALD analysis subset are provided in Supplementary Table 1.

**TABLE 1:** Baseline characteristics of children and adolescents in the Identification and Prevention of Dietary- and Lifestyle-induced Health Effects in Children and Infants/I.Family analysis sample grouped by short-term and habitual fruit and vegetable intake.

Characteristic	Short-term intake^1^			Habitual intake^2^	
	Fruit		Vegetables	Total fruit (*n* = 1788)	Total vegetables (*n* = 1788)
	Apple (*n* = 147)	Orange (*n* = 71)	Potato (*n* = 129)		
Intake (g/d)	130 (93–203)	130 (60–215)	120 (90–180)	165 (107–217)	110 (82–148)
Age (y)	8 (6–10)	9 (7–11)	9 (7–11)	9 (7–11)	
BMI *z*-scores^3^	0.16 (−0.54 to 0.68)	0.15 (−0.55 to 0.65)	0.094 (−0.46 to 0.58)	0.52 (−0.20 to 1.49)	
Sex					
Boys	72 (49)	33 (46)	60 (48)	945 (53)	
Girls	75 (51)	38 (54)	69 (52)	843 (47)	
Energy	1587 (1161–1945)	1645 (1317–1937)	1508 (1156–1865)	1634 (1495–1774)	
Country					
Italy	4 (3)	3 (4)	3 (2)	863 (48)	
Estonia	0 (0)	2 (3)	36 (28)	419 (23)	
Belgium	6 (4)	0 (0)	8 (6)	36 (2)	
Sweden	59 (40)	46 (65)	42 (33)	150 (8)	
Germany	71 (48)	15 (21)	36 (28)	180 (11)	
Hungary	2 (1)	0 (0)	3 (2)	90 (5)	
Spain	5 (3)	5 (7)	1 (1)	42 (2)	

Continuous values are presented as median ± IQR, and categorical data are presented as counts (%).

1 Short-term intake was defined and assessed for children and adolescents that reported the intake of these specific fruit or vegetables in 24-h dietary recalls (24h-DR) 1 or 2 d prior to the urine sample collection.

2 Habitual intake of fruit and vegetables was estimated by statistically combining data from 24h-DR and food frequency questionnaire using the National Cancer Institute method [27].

3 BMI was calculated by dividing body weight in kilograms by squared body height in meters and transformed to an age- and sex-specific z-score according to the study by Cole et al [42].

### Diet-metabolite associations in the IDEFICS/I.Family cohort

In this study, we incorporated both short-term and habitual intake measures to address the temporal complexity of diet-metabolite relationships. Short-term intake reflects immediate consumption and is more likely to align with metabolites that have rapid excretion kinetics, whereas habitual intake provides a broader view of consistent dietary patterns. This dual approach allows for a more nuanced interpretation of dietary biomarkers, particularly in free-living populations where both types of exposure may influence metabolomic signatures. Thus, in total, 59 molecular features were found to be significantly associated with fruit and vegetable intake and were subsequently subjected to compound annotation. Of these, 10 metabolites could be successfully annotated (Table 2) [[43], [44], [45], [46], [47], [48], [49], [50]], whereas the remaining unknown features are listed in Supplementary Table 2.

**TABLE 2:** Candidate biomarkers identified for fruit and vegetable intake in the Identification and Prevention of Dietary- and Lifestyle-induced Health Effects in Children and Infants/I.Family cohort (*N* = 1788).

Fruit/vegetables group	Intake type^1^	Metabolites	MSI^2^ level of confidence	Regulation	Coefficient	*P*^3^	HMDB ID	Metabolic pathway
Vegetables	Habitual intake	Hydroxyisovalerylcarnitine	2	Down	−0.001	0.017	—	Acylcarnitines [43]
Vegetables	Habitual intake	Octenoylcarnitine	2	Up	0.002	<0.001	—	Acylcarnitines [43]
Vegetables	Habitual intake	Tyrosine	1	Down	−0.003	0.014	0000158	Tyrosine metabolism [44]
Fruit	Habitual intake	Kynurenine^4^	1	Down	−0.001	0.003	0000684	Kynurenine metabolism [45]
Fruit	Habitual intake	5-Hydroxyindoleacetate	1	Up	0.001	0.001	0000763	Serotonin metabolism [46]
Fruit	Habitual intake	D-pantothenic acid	1	Up	0.001	0.008	0000210	β-Alanine metabolism [47]
Apple	Short-term/habitual intake	Hippuric acid	1	Up	0.001^5^	0.001	0000714	Benzoate metabolism [48]
Fruit						<0.001		
Vegetables						0.001		
Orange	Short-term intake	Ferulic acid 4-O-glucuronide	2	Up	0.002	0.005	0041733	Phenolic acids metabolism [49]
Orange	Short-term intake	Ferulic acid 4-O-sulfate	2	Up	0.003	<0.001	0029200	Phenolic acids metabolism [49]
Potato	Short-term intake	Gentisic acid	2	Up	0.001	0.02	0000152	Benzoate metabolism [50]

Fruit and vegetable intakes were modeled as the independent variables and covariates included age, sex, country, age- and sex-specific BMI *z*-scores, energy intake, dietary intake, and analytical batch.

Abbreviations: HMDB, Human Metabolome Database; MS, mass spectrometry; MSI, Metabolomics Standards Initiative.

1 Short-term intake was defined and assessed for children that reported the intake of these specific fruit or vegetables in 24-h dietary recalls (24h-DR) 1 or 2 d prior to the urine sample collection. The number of samples for short-term consumers was lower: *n* = 147 (apple), *n* = 71 (orange), and *n* = 129 (potato). Habitual intake of fruit and vegetables was estimated by statistically combining data from 24h-DR and food frequency questionnaire using the National Cancer Institute method and applied to all samples (*N* = 1788).

2 MSI, level 1, compounds identified by matching masses of MS/MS spectra and retention time to chemical standard; level 2, compounds putatively identified by matching of masses and MS/MS spectra to databases and literature [38].

3 *P* value by correction for multiple testing by false discovery rate using the Benjamini-Hochberg procedure.

4 In the additional wave-adjusted model, kynurenine was no longer statistically significant.

5 Coefficients of apple, fruit, and vegetables are all 0.001.

Positive associations were observed between habitual vegetable intake and octenoylcarnitine; habitual fruit intake and both 5-hydroxyindoleacetate and D-pantothenic acid; and both habitual fruit and vegetable intake and hippuric acid. In addition, positive associations were also observed between short-term orange intake and both ferulic acid 4-O-glucuronide and ferulic acid 4-O-sulfate, between short-term potato intake and gentisic acid, and between short-term apple intake and hippuric acid. Inverse associations were found between habitual vegetable intake with tyrosine and hydroxyisovalerylcarnitine, and habitual fruit intake with kynurenine.

### Diet-metabolite associations in the DONALD cohort

Apple, orange, total fruit, and total vegetable intake-metabolites associations were studied in the DONALD cohort with dietary intake from 3d-DWR and repeatedly collected 24-h urine samples of 297 children using a parallel analytical pipeline and statistical approach. In the DONALD cohort, the positive associations between habitual fruit as well as apple intake with hippuric acid were also found. Additional positive associations were observed in DONALD between orange intake and 3-hydroxyphenylacetate as well as xanthine, and fruit intake with 3-hydroxyphenylacetate. A negative association of apple intake with riboflavin was observed in the DONALD cohort. The results are presented in Supplementary Table 3.

### Validation of candidate biomarkers for fruit and vegetable intake

Table 3 depicts an overview of the validation process of the candidate biomarkers for fruit and vegetable intake. Hydroxyisovalerylcarnitine, tyrosine, and kynurenine were excluded for plausibility according to the criteria by Dragsted et al. [41] because food intake should increase the biomarker concentration. Octenoylcarnitine is not plausible to be a biomarker of vegetable intake since its precursor carnitine is less available in vegetables than in other foods [43]. D-pantothenic acid is ubiquitous in foods and not specific to fruit intake. The rest of the metabolites were plausible biomarkers according to criteria by Dragsted et al. [41] because their precursors are abundant in fruit and vegetables. In addition, the discovered plausible biomarkers showed increased intensity according to the amount of fruit and vegetables consumption, suggesting a potential dose-response relation with the intake of fruit and vegetables. Hippuric acid showed strong robustness and reproducibility to reflect habitual fruit intake in the 2 independent cohorts IDEFICS/I.Family and DONALD. Regarding the analytical aspects, quantification of 5-hydroxyindoleacetate (5-HIAA) [51] and hippuric acid [52] by HPLC-MS in urine is well established. At pH 4, 5-HIAA was stable in urine for ≤30 mo at −20°C, for 3 mo at 4°C, and for 3 wk at room temperature [53]. Hippuric acid is stable during sample handling (including storage at 4°C and −20°C) [54]. The time-response and reliability need further validation.

**TABLE 3:** Overview of the validation process and its application for the candidate biomarkers of fruit and vegetable intake.

Compound	Food group	Plausibility	Dose-response	Time-response	Robustness	Reliability	Stability	Analytical performance	Reproducibility
Hippuric acid	Apple/fruit	Y	Y	U	Y	U	Y	Y	Y
Hydroxyisovalerylcarnitine	Vegetables	N	Y	U	U	U	U	U	U
Octenoylcarnitine	Vegetables	N	Y	U	U	U	U	U	U
Tyrosine	Vegetables	N	Y	U	U	U	U	U	U
Kynurenine	Fruit	N	Y	U	U	U	U	U	U
5-Hydroxyindoleacetate	Fruit	Y	Y	U	U	U	Y	Y	U
D-pantothenic acid	Fruit	N	Y	U	U	U	U	U	U
Ferulic acid 4-O-glucuronide	Orange	Y	Y	U	U	U	U	U	U
Ferulic acid 4-O-sulfate	Orange	Y	Y	U	U	U	U	U	U
Gentisic acid	Potato	Y	Y	U	U	U	U	U	U

Plausibility: is the compound plausible as a specific biomarker for the food or food group (chemical/biological plausibility)? Dose-response: is there a dose-response relationship at relevant intake levels of the targeted food (quantitative aspect)? Time-response: is the biomarker kinetics described adequately to make a wise choice of sample type, frequency, and time window (time-response)? Robustness: has the marker been shown to be robust after intake of complex meals reflecting dietary habits of the targeted population (robustness)? Reliability: has the marker been shown to compare well with other markers or questionnaire data for the same food/food group (reliability)? Stability: is the marker chemically and biologically stable during biospecimen collection and storage, making measurements reliable and feasible (stability)? Analytical performance: is analytical variability (coefficient of variation %), accuracy, sensitivity, and specificity known to be adequate for at least one reported analytical method (analytical performance)? Reproducibility: has the analysis been successfully reproduced in another laboratory (reproducibility) [39]?

Abbreviations: N, no; U, unknown; Y, yes.

### Discrimination ability of hippuric acid for high compared with low apple and total fruit intake

**FIGURE 2:**
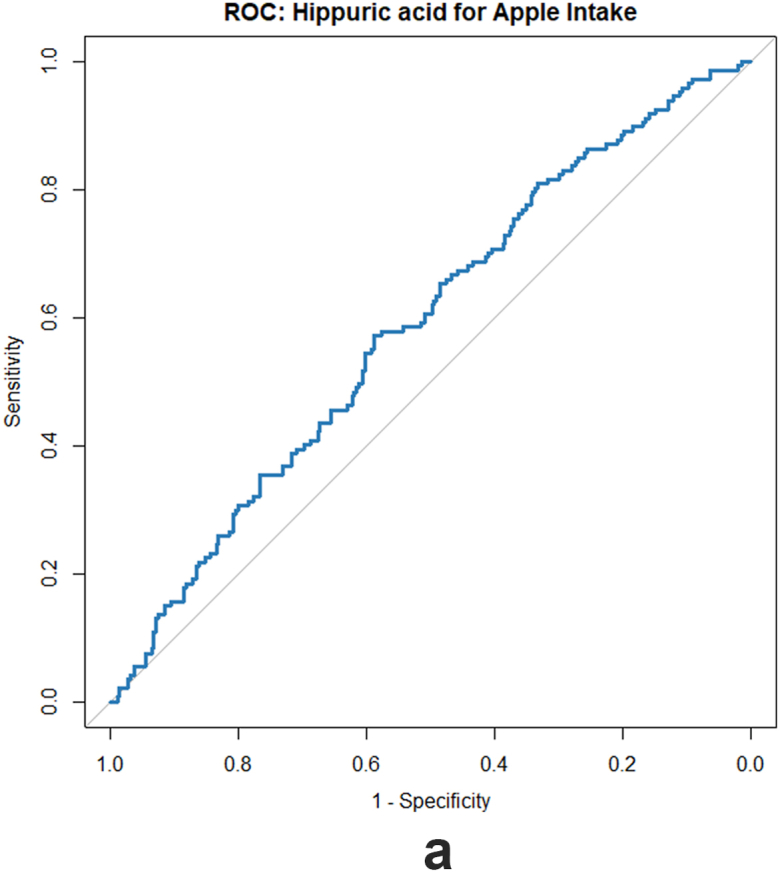

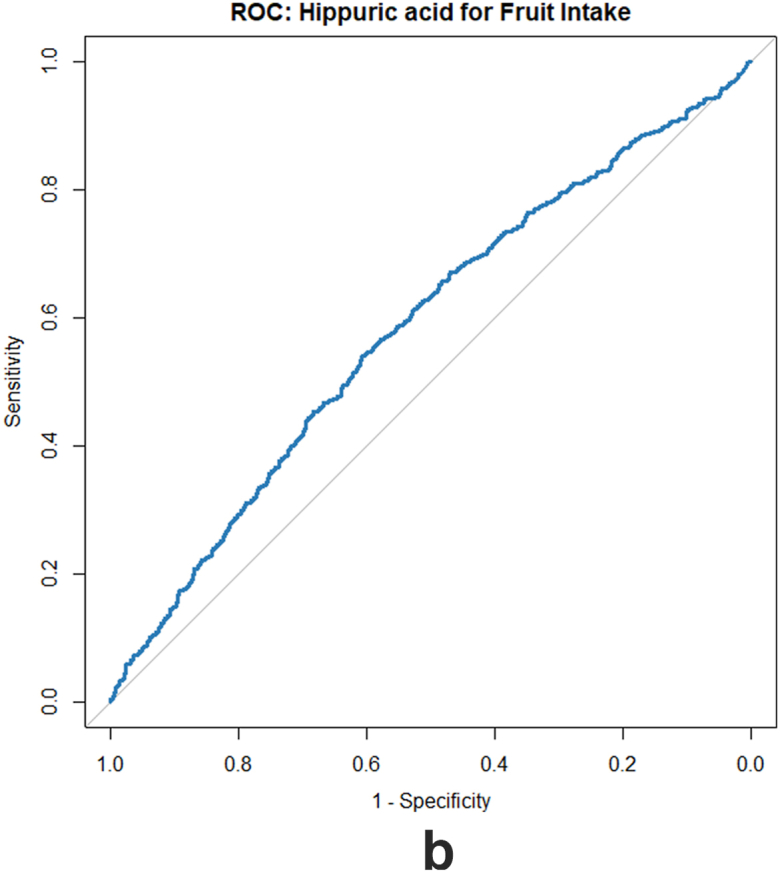
Discrimination ability of hippuric acid for high compared with low apple (A) and total fruit intake (B).

To assess the discriminatory ability of urinary hippuric acid in distinguishing between higher and lower apple (or total fruit) intake, we conducted an ROC analysis. The results indicated an AUC of 0.59 (95% CI: 0.53, 0.64) for apple consumption and an AUC of 0.58 (95% CI: 0.56, 0.61) for total fruit intake. These findings suggest that urinary hippuric acid offers low discriminatory information, but beyond random chance. The ROC curves are presented in Figure 2.

## Discussion

Our approach identified 10 metabolites that were strongly associated with fruit and vegetable intake, among which the association between apple and total fruit intake with hippuric acid was successfully replicated in the independent DONALD cohort.

The observed association between hippuric acid and fruit and vegetable intake is also consistent with previous studies conducted in both children [16,[55], [56]] and adults [[57], [58], [59]]. These findings suggest that urinary hippuric acid is a robust biomarker of fruit and vegetable intake across different populations, ethnic groups, and study design particularly for fruits. Although the ROC analysis showed low discrimination between higher and lower apple and total fruit intake, this performance is within the expected range for single-metabolite biomarkers evaluated against self-reported diet in free-living populations. These results further support the role of hippuric acid as a biomarker of fruit intake while also highlighting the inherent challenges and expected effect sizes in nutritional biomarker studies. Because children undergo rapid physiological changes that can influence urinary metabolite excretion, we additionally included examination wave as a covariate in a sensitivity analysis. After this adjustment, only a small number of unannotated features (*n* = 5) and 1 annotated metabolite (kynurenine) no longer met the statistical significance threshold. Importantly, all primary associations and overall effect patterns remained consistent. These findings indicate that developmental differences across waves exerted only a limited impact on the identified diet-metabolite associations.

However, the remaining associations were cohort specific and could not be replicated in the independent cohort. This limited reproducibility highlights the inherent challenges of nutritional metabolomics in pediatric populations. Several factors may contribute to these discrepancies. First, interindividual biological variability, such as differences in gut microbiota composition, enzymatic activity, and renal excretion capacity, can substantially influence metabolite profiles, even among individuals with similar dietary intake. Second, differences in dietary assessment methods—with FFQ and 24h-DR used in the IDEFICS/I.Family cohort compared with 3d-WDRs in DONALD—may result in variation in intake classification. Third, the urine sampling protocols differed between cohorts: morning spot urine was collected in IDEFICS/I.Family, whereas 24-h urine samples were collected in DONALD. This discrepancy may affect metabolite concentration and detectability due to diurnal variation and differences in dilution.

Additionally, population-level differences may have influenced the results. The IDEFICS/I.Family cohort includes participants from 7 European countries with diverse dietary habits and cultural food patterns, whereas the DONALD cohort includes only children from Germany. This geographic and cultural diversity could introduce variability in food choices, portion sizes, and food preparation methods, all of which impact the presence and concentrations of dietary metabolites. Together, these methodological and demographic differences underscore the difficulty of identifying universally applicable biomarkers across heterogeneous populations and highlight the importance of harmonized protocols and targeted validation in future studies.

The rest of the metabolites alone lack sufficient robustness to be considered as a unique biomarker, because they are not specific enough for fruit and vegetable intake, as discussed further. Although the potential usage for improving dietary intake assessment when combined with traditional dietary intake assessment methods may be worth further investigation. Some of the metabolites identified in the present study, for example, tyrosine and 5-hydroxyindoleacetate, have been previously associated with disease risk (e.g. diabetes and Alzheimer disease), as described further. They could be useful to further explore and interpret associations between fruit and vegetable intake and disease risk in future epidemiological studies.

### Gentisic acid

Gentisic acid is a secondary plant product, a derivative of phenolic acid. Gentisic acid exhibits an important role on human health, because of its anti-inflammatory, antigenotoxic, hepatoprotective, neuroprotective, antimicrobial, and especially antioxidant activities [60]. In the present study, urine gentisic acid was positively associated with potato intake. Although literature of gentisic acid content in potato is scarce except Karan et al. [61] who reported a certain potato genotype contains 30.79 ± 0.51 μg/kg. Future studies need to reassess the potential of gentisic acid as a possible biomarker of potato intake.

### Ferulic acid 4-O-glucuronide and ferulic acid 4-O-sulfate

Ferulic acid 4-O-glucuronide and ferulic acid 4-O-sulfate are derivatives of ferulic acid, which is a ubiquitous phenolic acid in plant tissues, such as grain bran, whole-grain food, fruit, and vegetables, and coffee [49]. Grains contain the highest amount of ferulic acid, followed by grapefruit and then orange. Owing to the antioxidant, antimicrobial, anti-inflammatory properties, ferulic acid is widely used in food industries. It is used as the raw material for the production of vanillin, and preservatives, for example, food gels, edible films, and sports foods [62]. In the present study, urinary ferulic acid 4-O-glucuronide and ferulic acid 4-O-sulfate showed a positive association with orange intake. The results are consistent with results of 2 intervention studies that found ferulic acid 4-O-glucuronide and ferulic acid 4-O-sulfate in urine after orange juice consumption [63,64]. Although, based on the abovementioned evidence, ferulic acid 4-O-glucuronide and ferulic acid 4-O-sulfate are not specific enough as biomarkers of orange intake.

### Hydroxyisovalerylcarnitine and octenoylcarnitine

Hydroxyisovalerylcarnitine and octenoylcarnitine belong to the class of acylcarnitines, which are the conjugation of fatty acids with carnitine. The biosynthesis of carnitine has been well documented that its major diet sources are meat, fish, and dairy products, whereas vegetables contain very little of carnitine [43]. Whereas, in the present study, hydroxyisovalerylcarnitine was found adversely associated with vegetable intake, octenoylcarnitine showed a positive association. Interestingly, octenoylcarnitine was found negatively associated with chocolate intake [65]. This may be better explained by endogenous metabolism rather than dietary intake. The adverse relationship between vegetable intake and hydroxyisovalerylcarnitine is also difficult to explain since the literature describing the catabolism of hydroxyisovalerylcarnitine in human subjects is scarce.

### Tyrosine

Tyrosine is a nonessential amino acid found in all plants and animals. In the present study, we found vegetable intake to be inversely associated with urinary tyrosine. Such findings may be attributable to residual or unmeasured confounding, correlated unmeasured lifestyle or metabolic factors. Given the inherent limitations of self-reported dietary intake, unexpected associations of this kind are not uncommon. Therefore, we refrain from providing mechanistic interpretations and emphasize that the signal requires validation in controlled dietary intervention studies.

### Kynurenine and 5-HIAA

Tryptophan is a crucial precursor of both serotonin and kynurenine metabolism. More than 95% of dietary tryptophan is metabolized via the kynurenine pathway, forming kynurenine and other derivatives [66], whereas ∼3% of tryptophan is used to synthesize serotonin throughout the body [46]. Serotonin is further metabolized to 5-HIAA as a terminal metabolite. An accelerated degradation of tryptophan with accompanying increase in kynurenine is often observed in many diseases and disorders, including neurodegeneration [45,67]. On the contrary, 5-HIAA was found to improve the pathophysiology and symptoms of Alzheimer disease in an animal model [68]. Indeed, circulating concentrations of 5-HIAA were recently reported to be inversely associated with dementia risk [69]. In our study, fruit intake was inversely associated with kynurenine and 5-HIAA. This may be a putative pathway of neuroprotective effects of fruit intake. Interestingly, plasma kynurenine:tryptophan ratio was positively associated with increased total fat intake, including total saturated fatty acid and total monounsaturated fatty acids intakes in a previous study [70]. Thus, it needs to be evaluated how the intake of other food groups and nutrients affects the urinary concentrations of kynurenine.

### D-pantothenic acid

Pantothenic acid, also known as vitamin B-5, is a water-soluble B-complex vitamin. D-pantothenic acid is the only biologically active form. Pantothenic acid is ubiquitous in foods, with high amounts in chicken, beef, whole grain, broccoli, eggs, cheese, lobster, and fruits also containing moderate amounts [47,71]. The adequate intake of pantothenic acid for children and adolescents is 4 and 5 mg/d, respectively, based on observed intakes in Europe [72]. In the present study, D-pantothenic acid showed a positive association with fruit intake, suggesting that fruit may be a relevant dietary source. However, pantothenic acid cannot be considered a candidate biomarker for fruit intake, due to lack of specificity.

Except for the fruit/vegetable-hippuric acid intake association, the other well-documented diet-metabolite associations in adults like proline betaine for citrus fruit intake [73] or phloretin for apple [74] could not be replicated in our study. The possible reason might be that although the metabolomics analysis in our study was untargeted, we may still have missed some metabolites. Another potential explanation could be that due to age, gut microbiota, and diet pattern differences, the postprandial metabolism between children and adults may not necessarily comparable [16]. Therefore, further studies regarding known biomarkers for fruit and vegetable intake in adults should be validated in child populations, for example, with targeted approaches or experimental designs.

### Limitations and strengths

Our study had several limitations. First, dietary intake data, including 24-h DR and FFQ were retrospectively self-reported, which could lead to recall errors and reporting bias, resulting in imprecise estimates of the true effect. We therefore replicated our results in another independent cohort with prospective dietary intake data from 3d-WDRs. The reproducibility of hippuric acid and apple/fruit intake association in both cohorts further suggested that our data are reliable. However, the limitations of replication may stem from differences in urine collection protocols (morning spot urine in IDEFICS/I.Family compared with 24-hour urine in DONALD) and population characteristics (multicountry compared with single-country cohort). These findings underscore the need for harmonized protocols in future studies.

Second, for short-term intake, the sample size of some groups was quite small because the time between 24h-DR and urine collection differed greatly between participants. We kept only the samples with 1- or 2-d difference for further analysis. For habitual intake, we have the data only for total fruit and total vegetable intake and not for individual species. This may cause the possibility of not finding promising dietary biomarkers. In addition, future studies may benefit from focusing on extreme intake contrasts—such as exceptionally high consumers compared with nonconsumers—which could improve signal detection in metabolomic analyses and support biomarker validation.

Third, the annotation of metabolite features remains a major challenge in untargeted metabolomics. Many of the molecular features associated with fruit and vegetable intake could not be conclusively identified, potentially resulting in the loss of biologically relevant information. Nonetheless, we have made all putatively annotated and unknown features available in Supplementary Table S2 for future reference.

In terms of statistical methods, 1 limitation concerns the relatively high misclassification rates and modest *R*^2^ values observed in the MUVR-PLS and RF models. Model performance metrics are reported in Supplementary Table 4. However, this is not unexpected in nutritional metabolomics, particularly when attempting to distinguish consumers from nonconsumers based on urinary profiles. The modest classification performance likely reflects the low signal-to-noise ratios of metabolite responses to episodic dietary intake, as well as class imbalance due to a high proportion of nonconsumers in the data set. Additionally, permutation testing showed that for 4 of the 5 food groups (apple, orange, total fruit, and total vegetables), permutation *P* values were < 0.05. This indicates that the observed cross-validated model performance was significantly higher than would be expected by chance for the representative models examined (Supplementary Figure 1), supporting the presence of genuine—albeit modest—underlying signal. It is important to note that the primary contribution of the MUVR-PLS and MUVR-RF models in this study lies in variable screening and the identification of candidate biomarkers, rather than in providing precise dietary intake prediction tools. Given the complexity of nutritional metabolomics data, modest *R*^2^ values and imperfect classification accuracy are expected and do not undermine the utility of these models for robust feature selection. The repeated double cross-validation framework of MUVR is specifically designed to minimize overfitting and to identify stable, reproducible metabolite signals even when predictive performance is limited. Thus, the outputs of these models should be interpreted primarily as data-driven indicators of plausible biomarker candidates that warrant further validation, rather than as tools capable of accurately estimating dietary intake at the individual level. The features identified were subsequently evaluated using mixed-effects regression models, which provided more interpretable estimates of dietary associations. Finally, although we applied robust cross-validation procedures and controlled for multiple hypotheses testing using FDR correction, the use of semiquantitative, unitless metabolomics data limits the generalizability of absolute effect sizes. In addition, although we adjusted for key confounding variables (e.g. age, sex, BMI *z*-score, country, energy intake, and analytical batch), the possibility of residual confounding due to unmeasured factors cannot be fully excluded.

To our knowledge, this is the first study to investigate the food metabolome in a large international children cohort. We used an exploratory approach with untargeted metabolomics analysis using sophisticated UPLC-MS, which allowed the coverage of small metabolites of high polarity or volatility. Owing to the nature of data that have large numbers of variables and few observations, 2 advanced statistical methods RF and PLS were conducted with the R package MUVR with a nested repeated double cross-validation procedure to reduce the risk of model overfitting. Lastly, a key strength of this study is the availability of independent data from the DONALD cohort, which included both 3d-WDRs and repeated 24-h urine collections. This enabled the application of the same analytical and statistical pipeline to replicate our findings.

In conclusion, We were able to identify plausible biomarkers of fruit and vegetable intake in free-living child and adolescence population, of which the association between fruit and vegetable intake and hippuric acid was in line with previous studies. These findings suggest that hippuric acid may enhance the objective assessment of fruit and intake in children and adolescents. In addition, this limited replication underscores the inherent challenges of nutritional metabolomics in pediatric populations while also reflecting both the potential and the limitations of observational study designs. These findings emphasize the need for further validation in well-controlled settings.

## Author contributions

The authors’ responsibities were as follows — AF, UN, IH, PKR, JR, KO: designed the research; SDH, MH, IH, LL, DM, LAM, PR, TV, SS, MW, and WA: conducted the IDEFICS/I.Family cohort; JR, MM, DA, PKR: conducted metabolomics analyses including molecular features identification and annotation; LY: wrote the paper; SM: conducted the statistical analysis in replication study; KO: revised and supervised the statistical analysis in replication study; LY, SM, JG, RF: performed or made significant contributions toward the statistical analysis; LY, SM, JG, JR, RF, MW, SDH, MH, IH, LL, DM, LAM, PR, TV, WA, AF, UN, PKR, KO: edited the draft; YL, AF: had primary responsibility for final content; and all authors: have read and approved the final manuscript.

## Data availability

Data described in the manuscript, code book, and analytic code will be made available upon request pending application and approval.

## Funding

This study was funded by the German Research Foundation (DFG project number 406710821) and the Agence Nationale de la Recherche (ANR project number ANR-18-CE92-0060). This work was done as part of the IDEFICS (http://www.idefics.eu) and I.Family studies (http://www.ifamilystudy.eu/) and the DONALD study. This study was also funded by the European Commission within the Sixth RTD Framework Programme contract number 016181 (FOOD) and the Seventh RTD Framework Programme contract number 266044 for IDEFICS/I.Family. The DONALD study is financially supported by the Ministry of Science and Research of North Rhine-Westphalia, Germany. This study was also funded by the Chinese Scholarship Council (CSC number 202009370100).

## Conflict of interest

The authors report no conflicts of interest. Where authors are identified as personnel of the International Agency for Research on Cancer/World Health Organization, the authors alone are responsible for the views expressed in this article and they do not necessarily represent the decisions, policy or views of the International Agency for Research on Cancer/WHO.
