# Supplementary material for: Exploratory Screening and Replication of Urinary Biomarkers of Fruit and Vegetable Intake in Free-living European Children and Adolescents Using Untargeted Metabolomics
Source: J Nutr. 2025 Dec 29;156(2):101302. doi: 10.1016/j.tjnut.2025.101302 (PMC12975373; doi:10.1016/j.tjnut.2025.101302)
Supplement: multimedia component 1 [file mmc1.docx]

**Exploratory screening and validation of fruit and vegetables intake biomarker in free-living European children and adolescents using untargeted metabolomics**

Li Yuan ^1^, Samuel Muli ^2^, Jantje Goerdten ^1^, Jodi Rattner ^3^, Mira Merdas ^3^, David Achaintre ^3^, Ronja Foraita ^1^, Maike Wolters ^1^, Stefaan De Henauw ^4^, Monica Hunsberger ^5^, Inge Huybrechts ^3^, Lauren Lissner ^5^, Dénes Molnár ^6^, Luis A. Moreno ^7,8^, Paola Russo ^9^, Toomas Veidebaum ^10^, Wolfgang Ahrens ^1^, Ute Nöthlings ^2^, Pekka Keski-Rahkonen ^3^, Kolade Oluwagbemigun ^2^, Anna Floegel ^1,11^

^1^Leibniz Institute for Prevention Research and Epidemiology – BIPS, Bremen, Germany

^2^Unit of Nutritional Epidemiology, Department of Nutrition and Food Sciences, University of Bonn, Bonn, Germany

^3^International Agency for Research on Cancer (IARC), Lyon, France

^4^Department of Public Health and Primary Care, Ghent University, Ghent, Belgium

^5^School of Public Health and Community Medicine, Institute of Medicine, Sahlgrenska Academy, University of Gothenburg, Gothenburg, Sweden

^6^Department of Pediatrics, Medical School, University of Pécs, Pécs, Hungary

^7^GENUD (Growth, Exercise, NUtrition and Development) Research Group, Faculty of Health Sciences, University of Zaragoza, Instituto Agroalimentario de Aragón (IA2) and Instituto de Investigación Sanitaria Aragón (IIS Aragón), Zaragoza, Spain

^8^Consorcio CIBER, M.P. Fisiopatología de la Obesidad y Nutrición (CIBERObn), Instituto de Salud Carlos III (ISCIII), Madrid, Spain

^9^Institute of Food Sciences, CNR, Avellino-Italy

^10^National Institute for Health Development, Tallinn, Estonia

^11^Section of Nutrition and Dietetics, Faculty of Agriculture and Food Sciences, Hochschule Neubrandenburg - University of Applied Sciences, Neubrandenburg, Germany

**Laboratory analysis for IDEFICS/I.Family**

**Sample Preparation and randomization**

Urine samples (n=1800) were shipped from the central biobanks in Bremen, Germany, to the laboratory at the International Agency for Research on Cancer (IARC, Lyon, France) for metabolomics analyses. The study samples were anonymised and randomised prior to shipment. The repeated samples for each study participant were analysed next to each other in random order, and sample pairs were randomised across the batch. Randomization of the samples was further stratified by country for the multicentre IDEFICS/I.Family cohort samples, to present an equal proportion of samples from each country on each plate.

Urine samples were prepared by diluting 30 uL of urine with ultra-pure water based on normalization to lowest specific gravity in all samples (IDEFICS: 1.008). Then 30 uL of the diluted urine samples were mixed with 270 uL of cold acetonitrile in Agilent Captiva 96 Deep Well plates (Agilent Technologies France; ref: A696001000B). The precipitate was filtered and 100 uL was transferred to Thermo Well 96 plates (Thermo Electron SAS; ref: 6820-4100). The plate was immediately sealed with a rapid EPS adhesive plate sheet (Teknolab Sorbent;ref: BC-REPS001) and analysed. Quality control (QC) samples were prepared from a sample pool that was made by mixing small aliquots of all samples and extracted along with the study samples. Blank samples were also prepared along the urine samples in an identical manner, only leaving out urine in the process. Each well plate included four individually prepared QCs and two blanks. The study samples were anonymised and randomised prior to shipment. The repeated samples for each study participant were analysed next to each other in random order, and sample pairs were randomised across the batch. Randomization of the samples was further stratified by country for the multicentre IDEFICS/I.Family cohort samples, to present an equal proportion of samples from each country on each plate.

**Sample Analysis**

Samples were analysed as 10 independent analytical batches consisting of 2 individual 96-well plates. The repeated samples were analysed next to each other in random order, and sample pairs were randomized across the batch. A UHPLC-QE-MS system was used that consisted of a Dionex UltiMate 3000 Binary LC system, and a Q-Exactive mass spectrometer with heated electrospray ionization (HESI-II) (Thermo Scientific). Samples were kept at 5°C and 2 µL was injected. An ACQUITY UHPLC HSS T3 column (2.1 × 100mm, 1.8 μm; Waters) was used at 45 °C and the mobile phase consisted of ultrapure water and LC-MS grade methanol, both with 0.05 % (v/v) of formic acid. The gradient profile was as follows: 0–6 min: 5% to 100% methanol, 6–10.5 min: 100% methanol, 10.5–13 min: 5% methanol. The flow rate was 0.4 ml/min.

The mass spectrometer was operated in a positive/negative switching polarity using the following conditions: spray voltage 4.0 kV, sheath gas flow rate 50 (Arbitrary unit; A.u), auxiliary gas flow rate 13 (A.u), sweep gas flow rate 3 (A.u), Aux gas heater temperature 425°C, capillary temperature 260°C and a S-Lens RF level 60%. For the analysis a full MS scan mode over a mass range of 66.7 to 1000 Da, at a resolution 35000 with an associated scan rate at 2.1Hz. AGC target 1e6 and a maximum injection time 50 ms was applied. MS/MS analyses were performed on QC samples with an isolation width of 2.0 Da, in positive and negative modes at 3 normalized collision energies 30, 60 and 90. Data was acquired in centroid format.

**Data Processing**

Pre-processing was performed using Compound Discoverer 3.3 software (Thermo Fisher Scientific). A minimum peak intensity threshold and mass tolerance of 500,000 and 5 ppm respectively were used to find [M+H]^+^ and [M-H]^-^ ions in positive and negative mode data, respectively. Feature alignment between samples was performed with maximum retention time window of 0.05 min and mass tolerance of 5ppm. Features were put forward into the feature table only if they were present in at least 2% of the overall samples. Features present in every blank sample were excluded, unless 5-fold greater in average intensity in samples. Peak areas were used as a measurement of intensity.

**Quality Control**

Quality control was performed using data from the QC samples. The assessment was based on the following attributes:

- Response stability: in chronological order, area median response of features found in all QC samples (Chart 1)
- Response variability: distribution of MS features according to their Relative Standard Deviations (RSD%) of features found in all QC samples (Chart 2)
- Response variability: RSD% of 10 known compounds in all QC samples (Data Table 1 and 2)

**Identification of metabolites**

The features indicated by the statistical analysis as significant (molecular features associated with fruit and vegetable intake were selected by multiple statistical approaches) were grouped by retention time (with a tolerance of 0.05 min) and intensity using Spearman correlation across all samples to help in finding features originating from the same compound, requiring a minimum of correlation coefficient of 0.75. The features were compared with the in-house database of analytical standards with 10 ppm molecular weight and 0.25 min retention time tolerance and search of the m/z values against the Human Metabolome Database (HMDB) [1] with a 10 ppm mass tolerance, considering [M + H]+, [M + Na]+, and [M -H2O+H]+ adducts in positive mode and [M -H]-, [M +FA-H]-, and [M -H2O-H]- in negative mode.

The quality of the chromatographic peaks and spectra was inspected, and the plausibility of database candidates was assessed based on retention time, isotope pattern, adduct formation and neutral losses. The best matching identities were confirmed by MS/MS spectra of standard MS/MS. When standards are not available, MS/MS spectra were compared against those in mzCloud (www.mzcloud.org) or METLIN (www.metlin.scripps.edu) [2]. The level of identification was determined as proposed by Sumner et al. [3].


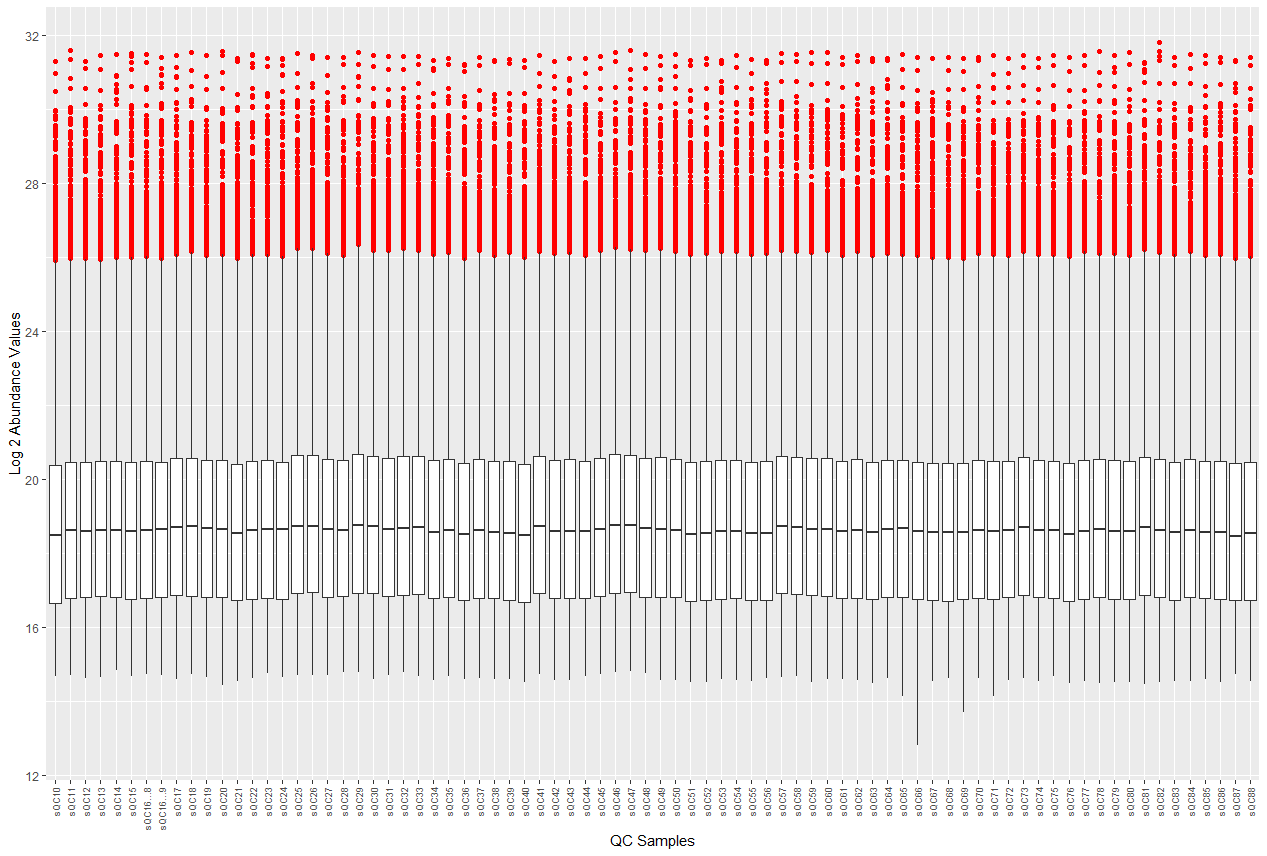


a


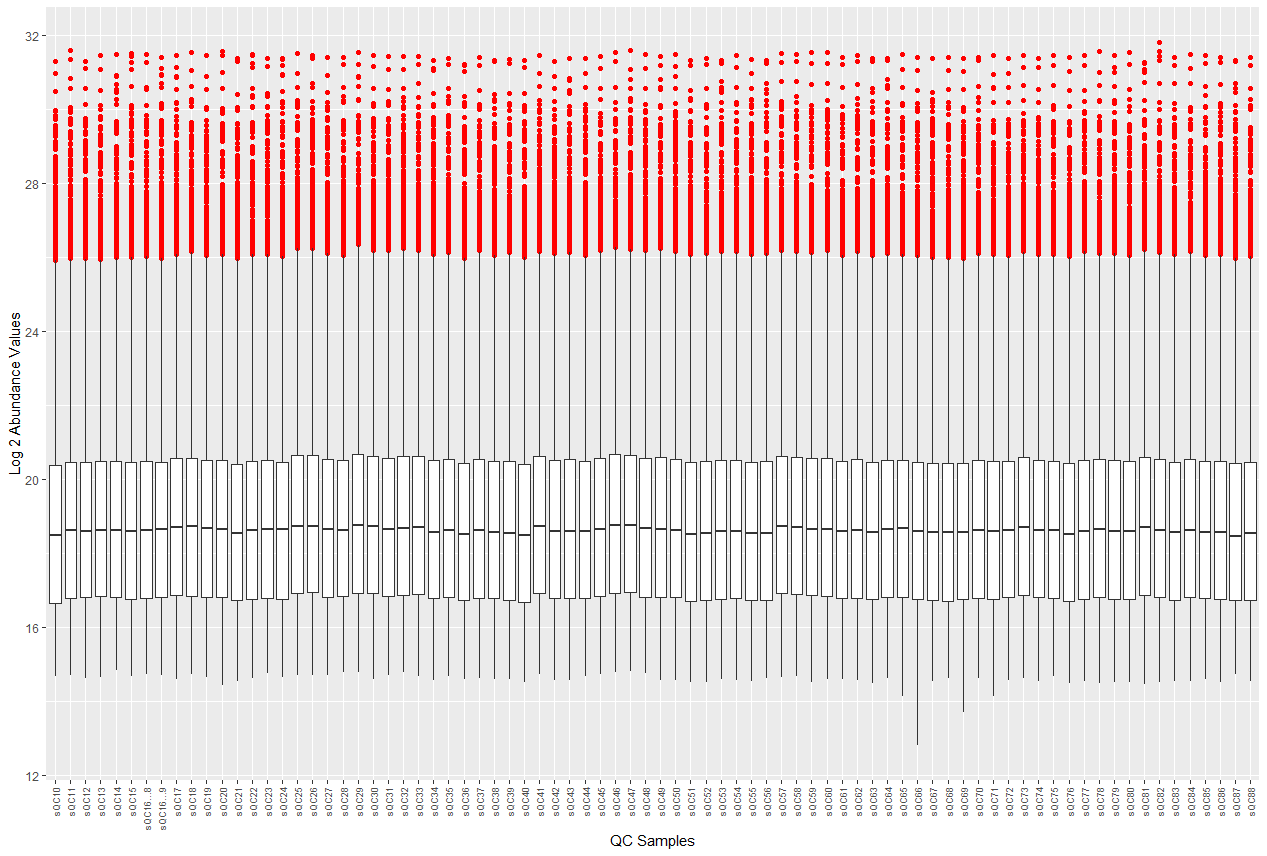


**b**

**Chart 1**. Log2 normalized intensity distribution plots for all features found in Positive Ionization Mode (a) and Negative Ionization Mode (b) of all QCs in IDEFICS cohort. Each batch contained two plates, and each plate contained four QC samples. Boxplots: median with 25^th^ and 75^th^ percentiles. Outliers in red. Samples in Chronological order from left to right

| a | b |
| --- | --- |

**Chart 2.** A variability histogram for the features found in Positive Ionization mode (a) and Negative mode (b) of all QC samples in IDEFICS/I.Family analysis (n=80).

**Data** T**able 1.** Variability of 10 known compounds in Positive Ionization mode across all QC samples in IDEFICS/I.Family analysis.

| Name | N | Area | %CV |
| --- | --- | --- | --- |
| Creatine | 80 | 699696753.400 | 4.94% |
| Glutamine | 80 | 27863050.440 | 10.49% |
| Hippuric acid | 80 | 1458472312 | 5.67% |
| Histidine | 80 | 26687713.510 | 9.81% |
| Hypoxanthine | 80 | 3593776.478 | 7.96% |
| Indole-3-acetic acid | 80 | 50209878.840 | 6.33% |
| Kynurenine | 80 | 17977013.840 | 8.24% |
| Phenylalanine | 80 | 366075651.800 | 6.90% |
| Tryptophan | 80 | 245166100.800 | 7.75% |
| Tyrosine | 80 | 105853986.200 | 7.35% |

**Data Table 2.** Variability of 10 known compounds in Negative Ionization mode across all QC samples in IDEFICS/I.Family analysis.

| Name | N | Area | %CV |
| --- | --- | --- | --- |
| Caffeine | 80 | 33711960.700 | 5.10% |
| Glutamine | 80 | 27909608.790 | 10.43% |
| Histidine | 80 | 26677649.590 | 9.87% |
| Hypoxanthine | 80 | 3603799.562 | 7.79% |
| Indole-3-acetic acid | 80 | 24727970.390 | 6.23% |
| Indolelactic acid | 80 | 3098682.583 | 7.90% |
| Lysine | 80 | 38957516.140 | 7.10% |
| Tryptophan | 80 | 244724778.500 | 7.64% |
| Tyrosine | 80 | 105691351.500 | 7.29% |
| Valine | 80 | 86391271.490 | 10.67% |

**Laboratory analysis for DONALD**

**Sample Preparation**

Samples (n=600) were prepared by diluting 30 uL of urine with ultra-pure water based on normalization to lowest specific gravity in all samples (DONALD: 1.079). Then 30 uL of the diluted urine samples were mixed with 270 uL of cold acetonitrile in Agilent Captiva 96 Deep Well plates (Agilent Technologies France; ref: A696001000B). The precipitate was filtered and 100 uL was transferred to Thermo Well 96 plates (Thermo Electron SAS; ref: 6820-4100) . The plate was immediately sealed with a rapid EPS adhesive plate sheet (Teknolab Sorbent; ref: BC-REPS001) and analysed. Quality control (QC) samples were prepared from a sample pool that was made by mixing small aliquots of all samples and extracted along with the study samples. Blank samples were also prepared along the urine samples in an identical manner, only leaving out urine in the process. Each well plate included four individually prepared QCs and two blanks.

**Sample Analysis**

Samples were analysed as 4 independent analytical batches consisting of 2 individual 96-well plates. The repeated samples points were analysed next to each other in random order, and sample pairs were randomized across the batch. A UHPLC-QE-MS system was used that consisted of a Dionex UltiMate 3000 Binary LC system, and a Q-Exactive mass spectrometer with heated electrospray ionization (HESI-II) (Thermo Scientific). Samples were kept at 5°C and 2 µL was injected. An ACQUITY UHPLC HSS T3 column (2.1 × 100mm, 1.8 μm; Waters) was used at 45 °C and the mobile phase consisted of ultrapure water and LC-MS grade methanol, both with 0.05 % (v/v) of formic acid. The gradient profile was as follows: 0–6 min: 5% to 100% methanol, 6–10.5 min: 100% methanol, 10.5–13 min: 5% methanol. The flow rate was 0.4 ml/min.

The mass spectrometer was operated in a positive/negative switching polarity using the following conditions: spray voltage 4.0 kV, sheath gas flow rate 50 (Arbitrary unit; A.u), auxillary gas flow rate 13 (A.u), sweep gas flow rate 3 (A.u), Aux gas heater temperature 425°C, capillary temperature 260°C and a S-Lens RF level 60%. For the analysis a full MS scan mode over a mass range of 66.7 to 1000 Da, at a resolution 35000 with an associated scan rate at 2.1Hz. AGC target 1e6 and a maximum injection time 50 ms was applied. MS/MS analyses were performed on QC samples with an isolation width of 2.0 Da, in positive and negative modes at 3 normalized collision energies 30, 60 and 90. Data was acquired in centroid format.

**Data Processing**

Pre-processing was performed using Compound Discoverer 3.3 software (Thermo Fisher Scientific). A minimum peak intensity threshold and mass tolerance of 500 000 and 5 ppm respectively were used to find [M+H]^+^ and [M-H]^-^ ions in positive and negative mode data, respectively. Feature alignment between samples was performed with maximum retention time window of 0.05 min and mass tolerance of 5ppm. Features were put forward into the feature table only if they were present in at least 2% of the overall samples. Features present in every blank sample were excluded, unless 5-fold greater in average intensity in samples. The final feature table was exported as a .xlsx file, with FoodMetch ID (FM_ID) on row 1, acquisition order (Study_ID) on row 2, , specific gravity measurement (SPG) in row 3, dilution factor (Dilution_Factor) in row 4, batch assignment (Batch) on row 5, and feature IDs in column 1 from row 7 onwards. Peak areas were used as a measurement of intensity.

**Quality Control**

Quality control was performed using data from the QC samples. The assessment was based on the following attributes:

- Response stability: in chronological order, area median response of features found in all QC samples (Chart 3)
- Response variability: distribution of MS features according to their Relative Standard Deviations (RSD%) of features found in all QC samples (Chart 4)
- Response variability: RSD% of 10 known compounds in all QC samples (Data Table 3 and Data 4).

# Results


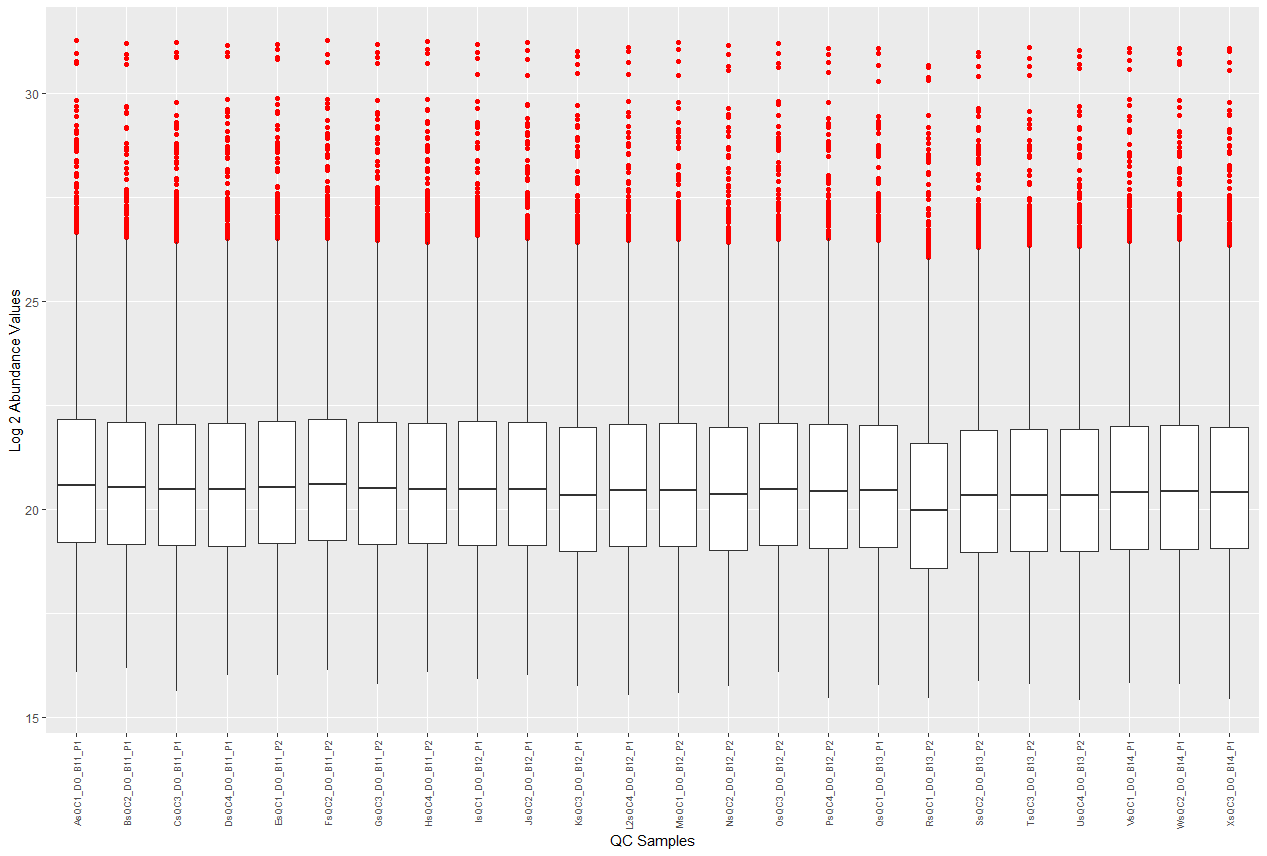


a


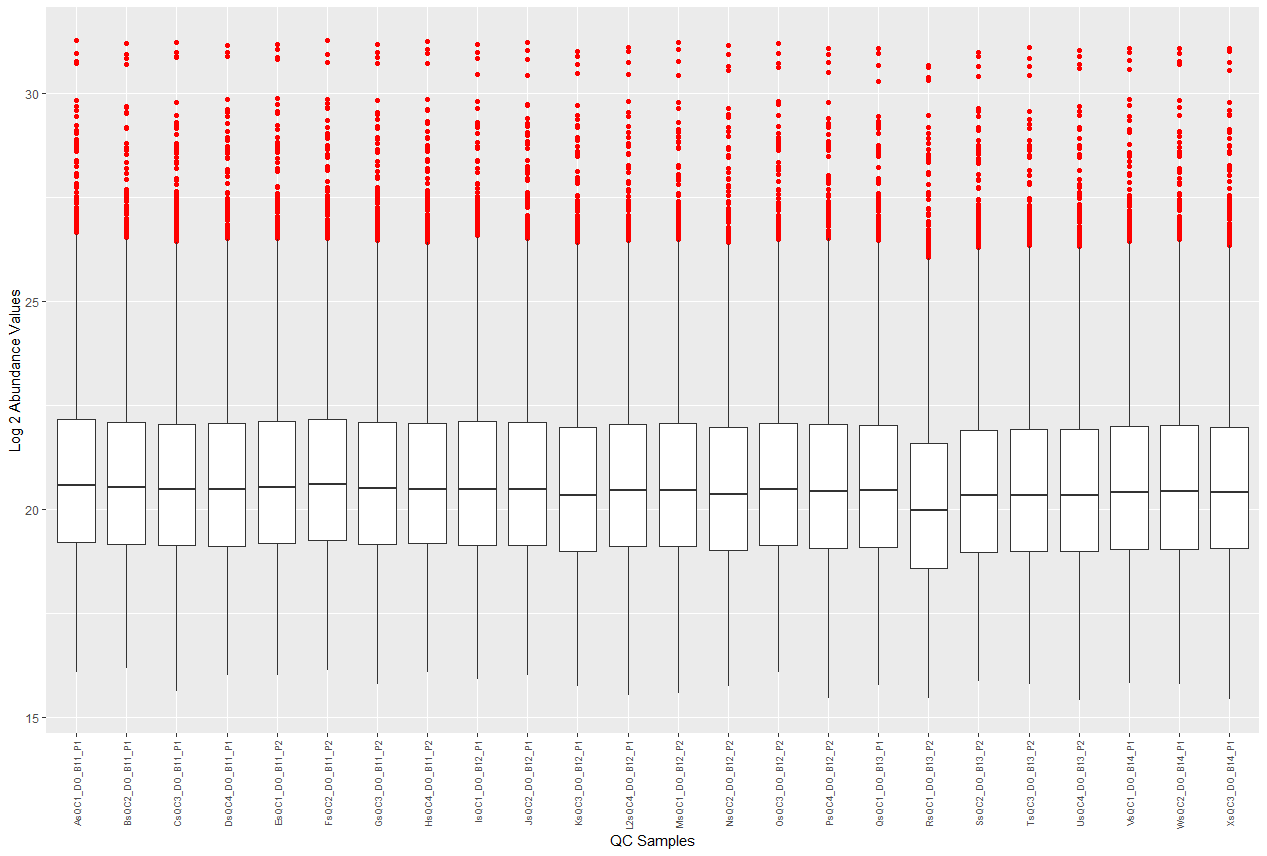


b

**Chart 3**. Log2 normalized intensity distribution plots for all features found in Positive Ionization Mode (a) Negative Ionization Mode (b) and of all QCs in DONALD cohort. Each batch contained two plates, and each plate contained four QC samples. Boxplots: median with 25^th^ and 75^th^ percentiles. Outliers in red. Samples in chronological order from left to right.

| 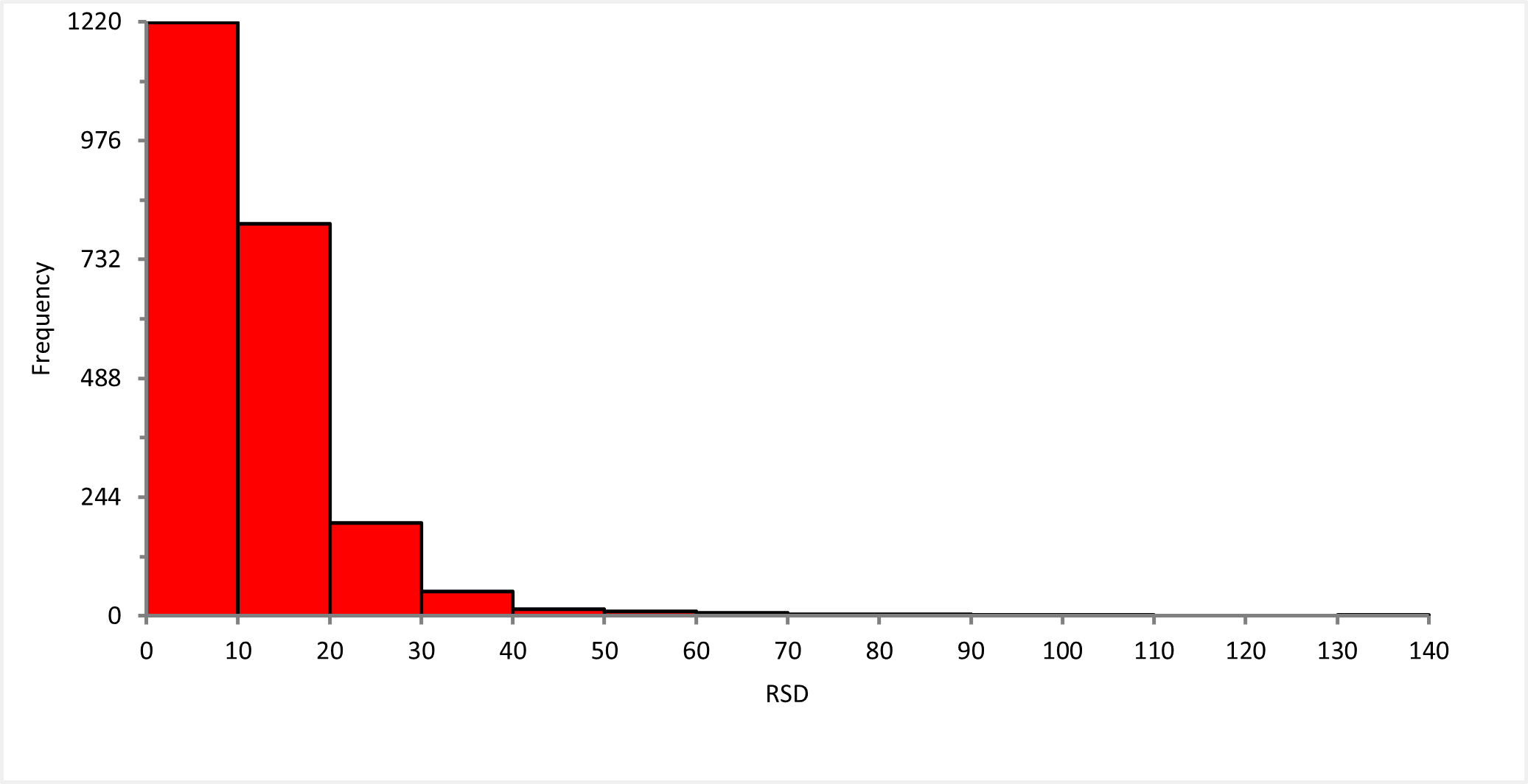  a | 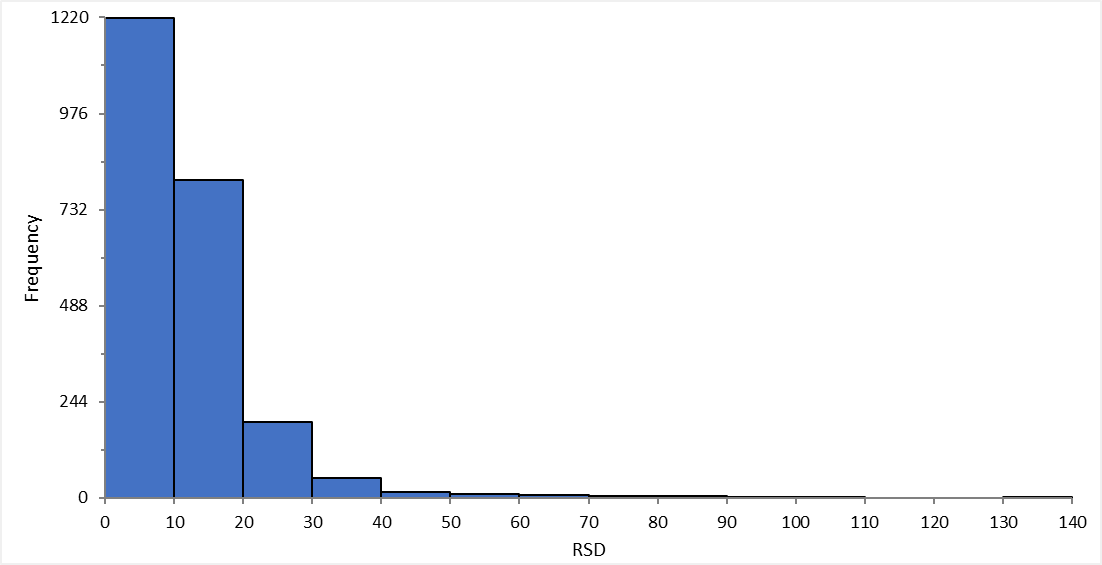  b |
| --- | --- |

**Chart 3.** A variability histogram for the features found in Positive Ionization mode (a) and Positive Ionization mode (b) of all QC samples in DONALD analysis (n=24).

**Data Table 3.** Variability of 10 known compounds in Positive Ionization mode across all QC samples in DONALD analysis.

| Name | N | Area | %RSD |
| --- | --- | --- | --- |
| Creatine | 24 | 853029237 | 7.60% |
| Glutamine | 24 | 20775211 | 6.77% |
| Hippuric acid | 24 | 1649345477 | 7.77% |
| Histidine | 24 | 15979430 | 8.39% |
| Hypoxanthine | 24 | 30080164 | 13.26% |
| Indole-3-acetic acid | 24 | 83278656 | 9.43% |
| Kynurenine | 24 | 9109718 | 11.36% |
| Phenylalanine | 24 | 253297831 | 10.41% |
| Tryptophan | 24 | 148977465 | 9.96% |
| Tyrosine | 24 | 79065675 | 10.44% |

**Data Table 4.** Variability of 10 known compounds in Negative Ionization mode across all QC samples in DONALD analysis.

| Name | N | Area | %CV |
| --- | --- | --- | --- |
| Caffeine | 24 | 33711961 | 5.10% |
| Glutamine | 24 | 20775211 | 6.77% |
| Histidine | 24 | 15979430 | 8.39% |
| Hypoxanthin | 24 | 3603800 | 7.80% |
| Indole-3-acetic acid | 24 | 83278657 | 9.43% |
| Indolelactic acid | 24 | 2466872 | 8.27% |
| Lysine | 24 | 51953917 | 5.99% |
| Tryptophan | 24 | 148977466 | 9.96% |
| Tyrosine | 24 | 9329105 | 11.01% |
| Valine | 24 | 61358423 | 5.84% |

**Statistical Analysis in DONALD**

MUVR with PLS modeling was used to select minimal optimal features related with respective fruit and vegetable intake (modeling parameters: nRep=50, varRatio = 0.90, nOuter = 8). For the linear mixed model molecular features were regressed on fruit and vegetable intake (continuous variables) with participant ID as random effect and adjusted for age, sex, BMI and total energy intake, while the batch effect correction was done as a pre-processing step, using the *ber* bagging method using the ‘dbnorm’ R package [4]. The Benjamini-Hochberg procedure to control the False Discovery Rate (FDR) at 5% was applied and accepting results with q < 0.05 as statistically significant.

**Supplementary Tables**

Table S1. Basic characteristics of children in the DONALD cohort analysis sample overall and by examination wave A and B

| **Characteristic** | **N** | **Overall**, N = 567^1^ | **A**, N = 297^1^ | **B**, N = 270^1^ |
| --- | --- | --- | --- | --- |
| **sex** | 567 |  |  |  |
| Male |  | 271 (48%) | 140 (47%) | 131 (49%) |
| Female |  | 296 (52%) | 157 (53%) | 139 (51%) |
| **Age at biosample collection (Years)** | 567 | 8 (3, 10) | 7 (3, 10) | 8 (4, 10) |
| **BMI z score^2^** | 567 | 0.10 (-2.30, 2.80) | 0.00 (-2.30, 2.70) | 0.1 (-2.30, 2.80) |
| **Fruit intake, g/d** | 567 | 278 (0, 1,337) | 260 (0, 1,337) | 292 (0, 1,042) |
| **Vegetables intake, g/d** | 567 | 70 (0, 468) | 65 (0, 386) | 76 (0, 468) |
| **Apple intake, g/d** | 567 | 118 (0, 908) | 122 (0, 749) | 105 (0, 908) |
| **Orange intake, g/d** | 567 | 8 (0, 1,096) | 7 (0, 1,096) | 10 (0, 1,027) |
| **Total energy intake, kcal/day** | 567 | 1,576 (687, 2,801) | 1,527 (823, 2,801) | 1,635 (687, 2,684) |

^1^n (%); Median (Range); ^2^ BMI z-scores is calculated based on German references of Kromeyer-Hauschild (2001) [5].

| Table S2 Overview of the features associated with fruit and vegetable intake without annotation in IDEFICS/I.Family. | | | | | | | | |
| --- | --- | --- | --- | --- | --- | --- | --- | --- |
| Fruit/vegetable group | Intake type | mode | Monoisotopic | Retention time | m/z^a^ | coefficient | P-value^b^ | Regulation |
| Apple | Acute | Neg | 148.03733 | 1.429 | 147.03005 | 0.002 | <0.001 | Up |
|  |  |  | 182.0579 | 2.752 | 181.05062 | 0.001 | 0.01 | Up |
|  |  |  | 400.17339 | 3.456 | 399.16611 | 0.002 | 0.004 | Up |
|  |  |  | 183.08968 | 3.709 | 182.0824 | 0.001 | 0.01 | Up |
| Orange | Acute | Pos | 143.0948 | 0.721 | 144.10208 | 0.006 | <0.001 | Up |
|  |  |  | 145.07412 | 0.955 | 146.0814 | 0.005 | <0.001 | Up |
|  |  |  | 145.07412 | 1.205 | 146.0814 | 0.004 | <0.001 | Up |
|  |  | Neg | 168.04265 | 1.516 | 167.03537 | 0.004 | <0.001 | Up |
|  |  |  | 143.05834 | 1.662 | 142.05106 | 0.005 | <0.001 | Up |
| Potato | Acute | Pos | 159.12607 | 0.744 | 160.13335 | 0.001 | 0.03 | Up |
|  |  |  | 146.06932 | 1.186 | 147.0766 | 0.001 | 0.02 | Up |
|  |  |  | 156.07888 | 3.308 | 157.08616 | 0.001 | 0.04 | Up |
|  |  |  | 400.17315^c^ | 3.418 | 401.18043 | 0.002 | 0.03 | Up |
|  |  | Neg | 265.09521 | 3.861 | 264.08793 | -0.002 | 0.018 | Down |
|  |  |  | 412.2099^c^ | 4.983 | 411.20262 | 0.002 | 0.02 | Up |
| Fruit | Usual | Pos | 142.98974^c^ | 0.526 | 143.99702 | -0.001 | 0.015 | Down |
|  |  | Neg | 161.06912 | 1.236 | 160.06184 | -0.001 | 0.04 | Down |
|  |  |  | 130.02691 | 1.511 | 129.01963 | 0.001 | <0.001 | Up |
|  |  |  | 217.09504 | 1.931 | 216.08776 | 0.001 | 0.01 | Up |
|  |  |  | 178.03016 | 1.969 | 177.02288 | -0.001 | 0.01 | Down |
|  |  |  | 280.10604 | 2.463 | 279.09876 | 0.001 | <0.001 | Up |
|  |  |  | 198.08907 | 4.229 | 197.08179 | -0.001 | 0.035 | Down |
| Vegetable | Usual | Pos | 135.06871 | 1.283 | 136.07599 | -0.001 | 0.02 | Down |
|  |  |  | 285.1212 | 1.618 | 286.12848 | 0.003 | <0.001 | Up |
|  |  |  | 120.02123 | 2.348 | 121.02851 | 0.001 | 0.01 | Up |
|  |  |  | 300.13218 | 2.706 | 301.13946 | 0.002 | <0.001 | Up |
|  |  |  | 245.16284 | 3.273 | 246.17012 | -0.001 | 0.002 | Down |
|  |  |  | 341.22022 | 3.698 | 342.2275 | 0.002 | <0.001 | Up |
|  |  |  | 256.14236 | 3.9 | 257.14964 | 0.002 | <0.001 | Up |
|  |  |  | 205.07418 | 4.063 | 206.08146 | 0.003 | <0.001 | Up |
|  |  |  | 130.0421 | 4.063 | 131.04938 | 0.003 | <0.001 | Up |
|  |  |  | 270.15797 | 4.452 | 271.16525 | 0.002 | <0.001 | Up |
|  |  |  | 199.12117 | 4.689 | 200.12845 | 0.002 | <0.001 | Up |
|  |  |  | 330.21952^c^ | 5.065 | 331.2268 | -0.001 | 0.005 | Down |
|  |  | Neg | 118.0268 | 1.371 | 117.01949 | 0.001 | <0.001 | Up |
|  |  |  | 118.0632 | 2.195 | 117.05588 | -0.001 | 0.01 | Down |
|  |  |  | 159.08982 | 2.292 | 158.08254 | -0.002 | <0.001 | Down |
|  |  |  | 249.0672 | 2.908 | 248.05992 | 0.002 | <0.001 | Up |
|  |  |  | 242.12673 | 3.372 | 241.11945 | 0.002 | <0.001 | Up |
|  |  |  | 256.14253 | 3.933 | 255.13525 | 0.002 | <0.001 | Up |
|  |  |  | 205.07379 | 4.096 | 204.06651 | 0.003 | <0.001 | Up |
|  |  |  | 202.02994^c^ | 4.153 | 201.02266 | 0.002 | <0.001 | Up |
|  |  |  | 270.15819 | 4.487 | 269.15091 | 0.002 | <0.001 | Up |
|  |  |  | 446.21543 | 4.821 | 445.20815 | 0.003 | <0.001 | Up |
|  |  |  | 214.12061 | 5.041 | 213.11333 | 0.003 | <0.001 | Up |
|  |  |  | 166.04875 | 0.698 | 165.04147 | 0.002 | <0.001 | Up |

^a^ m/z: mass to charge ratios. ^b^ p-value by correction for multiple testing by false discovery rate (FDR) using the Benjamini-Hochberg procedure. ^c^ In additional wave-adjusted model, these feature were no longer statistically significant.

Table S3 Potential biomarkers of fruit and vegetables intake in children of the DONALD cohort (n=567)

| **fruit/vegetable group** | **Metabolites** | **MSI^a^ Level** | **Regulation** | **Coefficient** | **p-value^b^** |
| --- | --- | --- | --- | --- | --- |
| Orange | Xanthine | 1 | up | 0.001 | 0.02 |
| Fruit | Hippuric acid | 1 | up | 0.002 | <0.001 |
| Oranges, Fruit | 3-hydroxyphenylacetate | 2 | up | 0.001 | <0.001 |
| Apple | Hippuric acid | 1 | up | 0.002 | <0.001 |
| Apple | Riboflavin | 2 | down | -0.001 | 0.03 |
| ^a^MSI Level: Metabolomics Standards Initiative. level 1, compounds identified by matching masses of MS/MS spectra and retention time to chemical standard; level 2, compounds putatively identified by matching of masses and MS/MS spectra to databases and literature. ^b^p-value by correction for multiple testing by false discovery rate (FDR) using the Benjamini-Hochberg procedure  Table S4: PLS and RF molecular feature selection in MUVR.   \| Mode \| \| Method \| Original variables \| Minimal-optimal variables \| Model performance ^a^ \| Permutation test  *p-*value ^b^ \| \| \| --- \| --- \| --- \| --- \| --- \| --- \| --- \| --- \| \| Apple \| \| \| \| \| \| \| \| \| pos \| MUVR-RF \| \| 390 \| 15 \| 53% misclassification \| - \| \| MUVR-PLS \| \| 390 \| 62 \| 54% misclassification \| - \| \| neg \| MUVR-RF \| \| 318 \| 21 \| 58% misclassification \| 0.04 \| \| MUVR-PLS \| \| 318 \| 51 \| 51% misclassification \| - \| \| Orange \| \| \| \| \| \| \| \| pos \| MUVR-RF \| \| 394 \| 8 \| 67% misclassification \| - \| \| MUVR-PLS \| \| 394 \| 91 \| 73% misclassification \| - \| \| neg \| MUVR-RF \| \| 331 \| 15 \| 82% misclassification \| - \| \| MUVR-PLS \| \| 331 \| 63 \| 71% misclassification \| 0.04 \| \| Potato \| \| \| \| \| \| \| \| pos \| MUVR-RF \| \| 386 \| 12 \| 76% misclassification \| - \| \| MUVR-PLS \| \| 386 \| 44 \| 70% misclassification \| 0.06 \| \| neg \| MUVR-RF \| \| 324 \| 12 \| 78% misclassification \| - \| \| MUVR-PLS \| \| 324 \| 37 \| 69% misclassification \| - \| \| Fruit \| \| \| \| \| \| \| \| pos \| MUVR-RF \| \| 378 \| 21 \| R^2^=0.07 \| - \| \| MUVR-PLS \| \| 378 \| 51 \| R^2^=0.12 \| - \| \| neg \| MUVR-RF \| \| 304 \| 23 \| R^2^=0.07 \| 0.04 \| \| MUVR-PLS \| \| 304 \| 48 \| R^2^=0.12 \| - \| \| Vegetable \| \| \| \| \| \| \| \| pos \| MUVR-RF \| \| 378 \| 21 \| R^2^=0.25 \| 0.04 \| \| MUVR-PLS \| \| 378 \| 61 \| R^2^=0.28 \| - \| \| neg \| MUVR-RF \| \| 304 \| 21 \| R^2^=0.25 \| - \| \| MUVR-PLS \| \| 304 \| 68 \| R^2^=0.29 \| - \|   ^a^ The model performance for short-term intake of apple, orange, and potato was evaluated with the percentage of consumer misclassification in the sample and coefficient of determination (R²) for habitual intake of fruit and vegetables. ^b^The statistical significance of differences in model performance between the actual and permuted models. Owing to the computational intensity of the procedure, one permutation run was performed per food group. -, owing to the computational intensity of the procedure, one permutation run was performed per food group.   \| 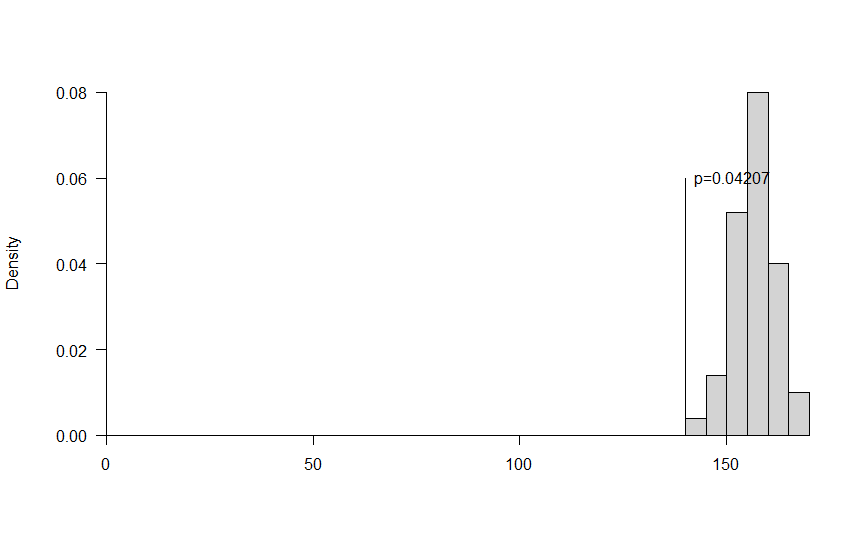  a \| 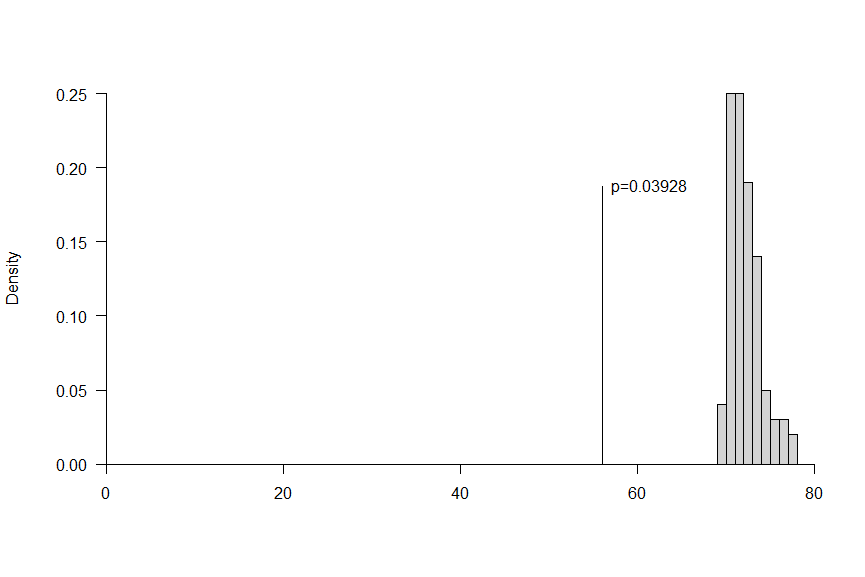  b \| \| --- \| --- \| \| 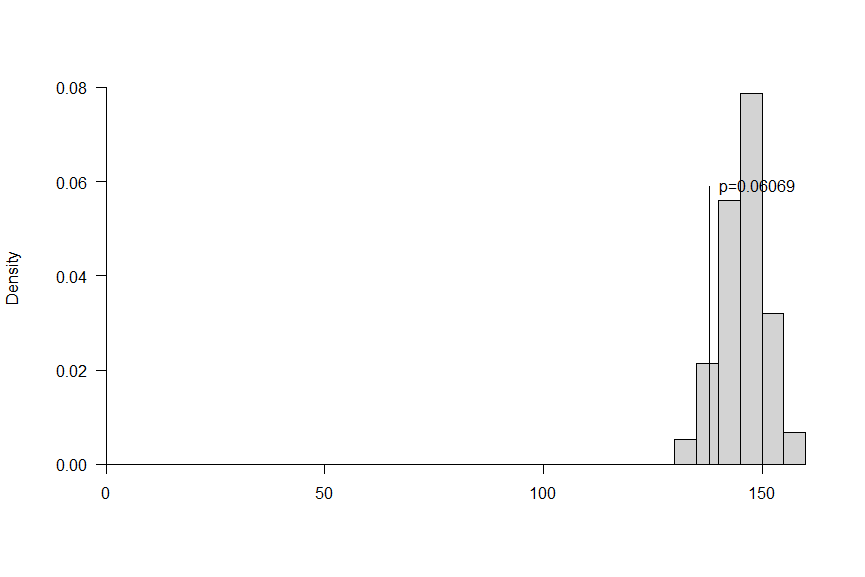  c \|  \| \| 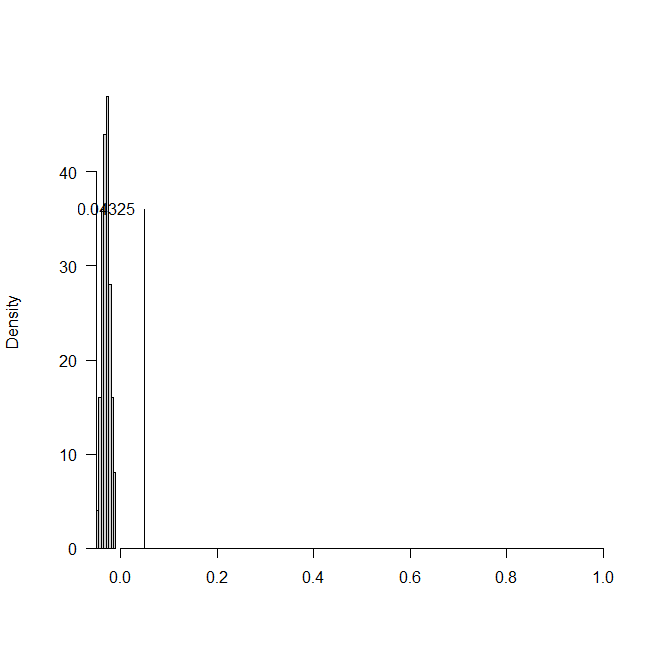  d \| 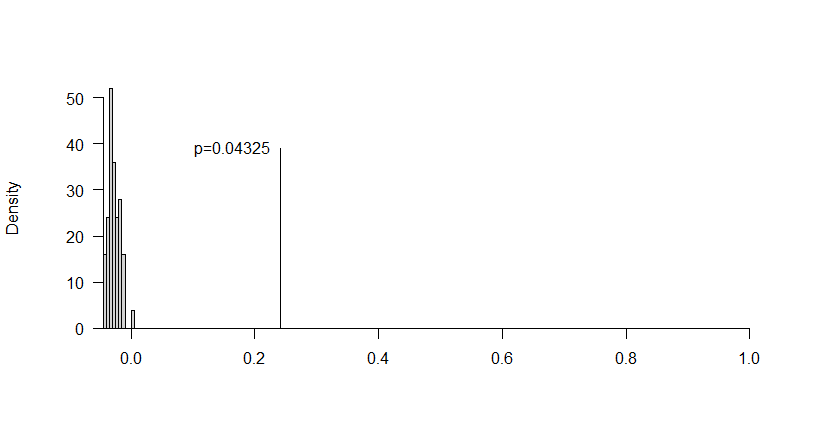  e \|   **Figure S1. Permutation distributions of cross-validated model performance for the MUVR models across fruit and vegetable groups**. Permutation tests (n = 100 iterations) were conducted by repeatedly randomizing outcome labels and recalculating model performance for each fruit and vegetable group. a (apple), b (orange), c (potato), d (fruit), e (vegetable). | | | | | |

**References**

1. Wishart, D.S., et al., *HMDB 4.0: the human metabolome database for 2018.* Nucleic Acids Res, 2018. **46**(D1): p. D608-d617.

2. Smith, C.A., et al., *METLIN: A Metabolite Mass Spectral Database.* 2005. **27**(6): p. 747-751.

3. Sumner, L.W., et al., *Proposed minimum reporting standards for chemical analysis.* Metabolomics, 2007. **3**(3): p. 211-221.

4. Bararpour, N., et al., *DBnorm as an R package for the comparison and selection of appropriate statistical methods for batch effect correction in metabolomic studies.* Sci Rep, 2021. **11**(1): p. 5657.

5. Kromeyer-Hauschild, K., et al., *Perzentile für den Body-mass-Index für das Kindes- und Jugendalter unter Heranziehung verschiedener deutscher Stichproben.* Monatsschrift Kinderheilkunde, 2001. **149**(8): p. 807-818.
